# Tuning the Nanoaggregates of Sialylated Biohybrid Photosensitizers for Intracellular Activation of the Photodynamic Response

**DOI:** 10.1002/chem.202100681

**Published:** 2021-05-17

**Authors:** Verónica Almeida‐Marrero, Marta Mascaraque, María Jesús Vicente‐Arana, Ángeles Juarranz, Tomás Torres, Andrés de la Escosura

**Affiliations:** ^1^ Department of Organic Chemistry / SIdI (MJVA) Universidad Autónoma de Madrid Campus de Cantoblanco 28049 Madrid Spain; ^2^ Departamento de Biología Universidad Autónoma de Madrid Campus de Cantoblanco 28049 Madrid Spain; ^3^ Instituto Ramón y Cajal de Investigaciones Sanitarias (IRYCIS) 28034 Madrid Spain; ^4^ Institute for Advanced Research in Chemistry (IAdChem) Campus de Cantoblanco 28049 Madrid Spain; ^5^ IMDEA Nanoscience Campus de Cantoblanco 28049 Madrid Spain

**Keywords:** biohybrids, nanoaggregates, photosensitizers, phthalocyanine, self-assembly

## Abstract

In the endeavor of extending the clinical use of photodynamic therapy (PDT) for the treatment of superficial cancers and other neoplastic diseases, deeper knowledge and control of the subcellular processes that determine the response of photosensitizers (PS) are needed. Recent strategies in this direction involve the use of activatable and nanostructured PS. Here, both capacities have been tuned in two dendritic zinc(II) phthalocyanine (ZnPc) derivatives, either asymmetrically or symmetrically substituted with 3 and 12 copies of the carbohydrate sialic acid (SA), respectively. Interestingly, the amphiphilic ZnPc‐SA biohybrid (**1**) self‐assembles into well‐defined nanoaggregates in aqueous solution, facilitating cellular internalization and transport whereas the PS remains inactive. Within the cells, these nanostructured hybrids localize in the lysosomes, as usually happens for anionic and hydrophilic aggregated PS. Yet, in contrast to most of them (e. g., compound **2**), hybrid **1** recovers the capacity for photoinduced ROS generation within the target organelles due to its amphiphilic character; this allows disruption of aggregation when the compound is inserted into the lysosomal membrane, with the concomitant highly efficient PDT response.

## Graphical abstract

**An insightful approach** towards activatable and nanostructured photosensitizers has been developed by preparing dendritic biohybrids composed of a core phthalocyanine decorated with multiple sialic acid moieties. The resulting hybrids self‐assemble into well‐defined nanoaggregates in aqueous solution, and disaggregate upon reaching the target organelles, resulting in the on‐site activation of a highly efficient photodynamic response.

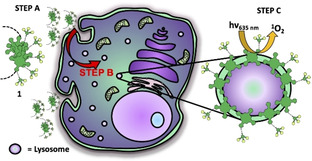

## Introduction

Porphyrinoids are second generation photosensitizers (PS) with a wide range of uses in nanotechnology and materials science,[^1^, ^2^] including molecular photovoltaics^[3]^ and biomedicine.^[4]^ Among them, phthalocyanines (Pc) are green/blue dyes comprised of four isoindole subunits, condensed into an aromatic macrocycle with an 18 π‐electron conjugated system. The photophysical and photochemical properties of Pc have been deeply investigated,[^5^, ^6^] showing high yields of production of singlet oxygen (^1^O_2_) and other reactive oxygen species (ROS), and a strong absorption band in the phototherapeutic window, which render them as excellent agents for PDT.[^7^, ^8^, ^9^] However, their low solubility and strong aggregation in aqueous media limit their medical applications.^[10]^ The therapeutic use of Pc also requires improving biocompatibility and cellular uptake,^[11]^ which can be achieved by conjugation with hydrophilic targeting units of biological origin, leading to so‐called photosensitizing biohybrid materials.[^4^, ^12^, ^13^, ^14^]

**Figure 1:**
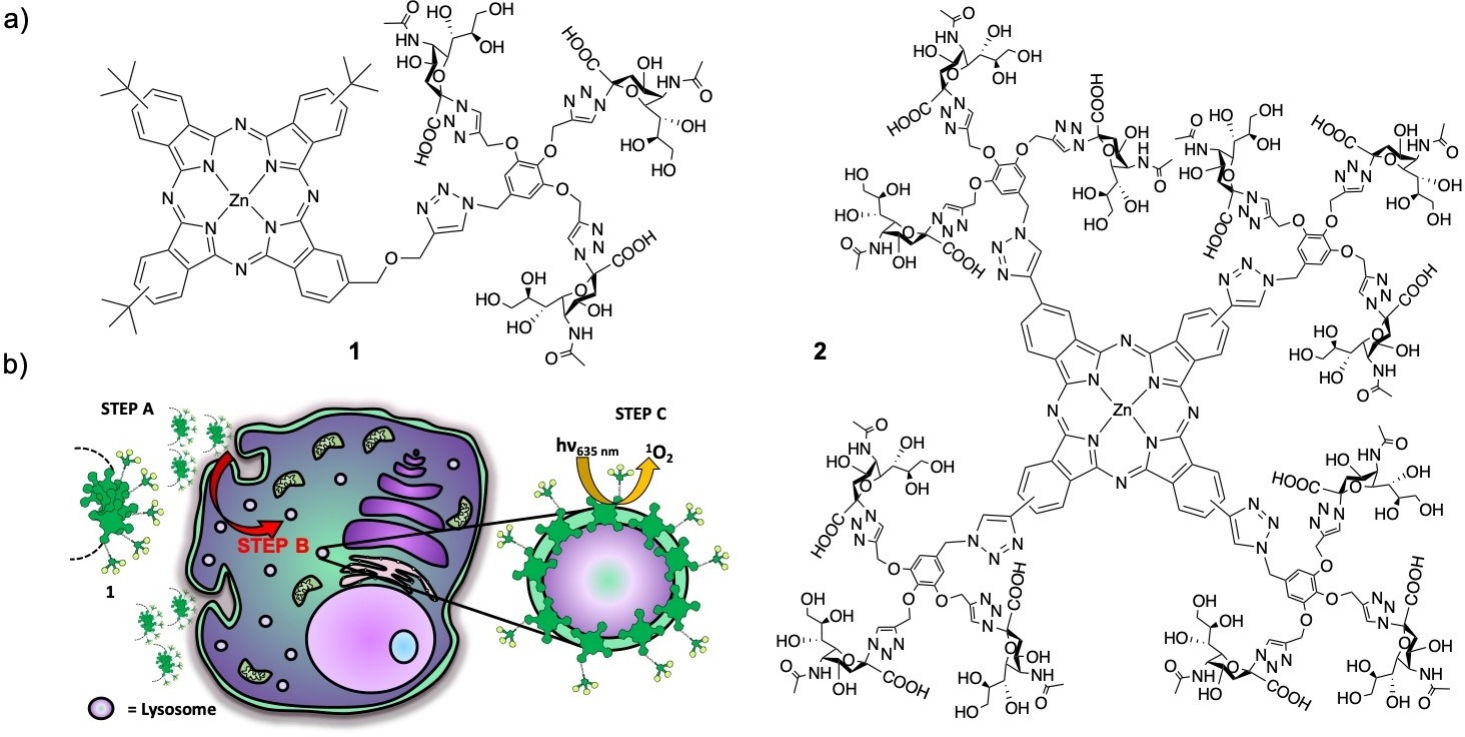
a) Structure of the dendritic ZnPc‐SA biohybrid PS **1** and **2**. b) Cartoon showing the strategy to enhance the photodynamic performance of **1**. The PS is inactive and highly aggregated into hydrophilic nanoparticles outside the cell (step A). Aggregation is disrupted after internalization, by incorporation into the membrane of lysosomes (step B, represented by red arrow), which results in an on‐site activation of the PS photodynamic properties (step C).

*N*‐Acetylneuraminic acid, most commonly known as sialic acid (SA), is a carbohydrate that plays an extensive role in the organism,^[15]^ and has been used for stabilization of nanocarriers towards cancer detection and targeting.[^16^, ^17^, ^18^, ^19^] Different biological/ synthetic polymers and colloidal nanoparticles with multiple SA units have demonstrated their surface‐protecting/masking properties, resulting in prolonged circulation times and enhanced pharmacokinetic parameters.[^20^, ^21^, ^22^, ^23^] Based on these advantageous properties, in this paper we describe a combined biohybrid strategy to tackle the issues that normally prevent an efficient photodynamic response of Pc against tumoral diseases. On one hand, decorating the ZnPc macrocycle with SA‐containing dendritic arms to ensure water‐solubility, biocompatibility and cell internalization. On the other hand, modulating the amphiphilic character and thus the aggregation behavior of the resulting ZnPc hybrids, to ensure an efficient ROS photogeneration once internalized. Such modulation results in a superior photodynamic performance of the asymmetrically substituted compound **1** (Figure 1), in experiments with three human superficial tumor cell lines: SCC‐13 (squamous cell carcinoma from face), A431 (squamous cell carcinoma from vulva), and HeLa (cervical adenocarcinoma), as they represent good models of the kind of tissues where this PS could be topically administered. This behavior is due to the PS self‐assembly into inactive, highly hydrophilic nanoaggregates in aqueous media, which facilitates cell penetration likely through endocytosis, and to disruption of aggregation once internalized, activating the PS when it gets inserted in the membrane of lysosomes within the cell (Figure 1b).

Carbohydrates are among the most promising biomolecular kinds that have been combined with Pc for biomedical applications, as they can improve the PS solubility and allow specific delivery to tumor cells.^[24]^ There are various strategies for the linkage of sugars to Pc dyes, which include axial and peripheral substitution of the macrocycle.[^25^, ^26^, ^27^] Different spacers can be used for this purpose,[^28^, ^29^, ^30^, ^31^] with the possibility to tune the number and distribution of carbohydrate moieties over the Pc scaffold. This can affect the PS amphiphilicity, while the incorporation of multiple units of the same sugar in dendritic nanostructures can help to achieve high water‐solubility and multivalency in the interaction with specific biological receptors.[^32^, ^33^, ^34^] On these bases, we have synthesized two dendritic ZnPc‐SA compounds **1** and **2** (Figure 1a), bearing either 3 or 12 SA moieties, respectively, which present different self‐assembling features and photodynamic properties in buffered aqueous media and in subcellular organelles. The specific behavior of **1** can be ascribed to its amphiphilic character, owing to the asymmetric pattern of sialylated substituents displayed on the Pc macrocyclic platform. The present work therefore reveals how the intimate relationship between self‐assembly and photochemical activity induced by the PS in the subcellular medium where it is localized determines the observed photodynamic response.

## Results and Discussion

### Synthesis of dendritic ZnPc‐SA biohybrids

**Scheme 1:**
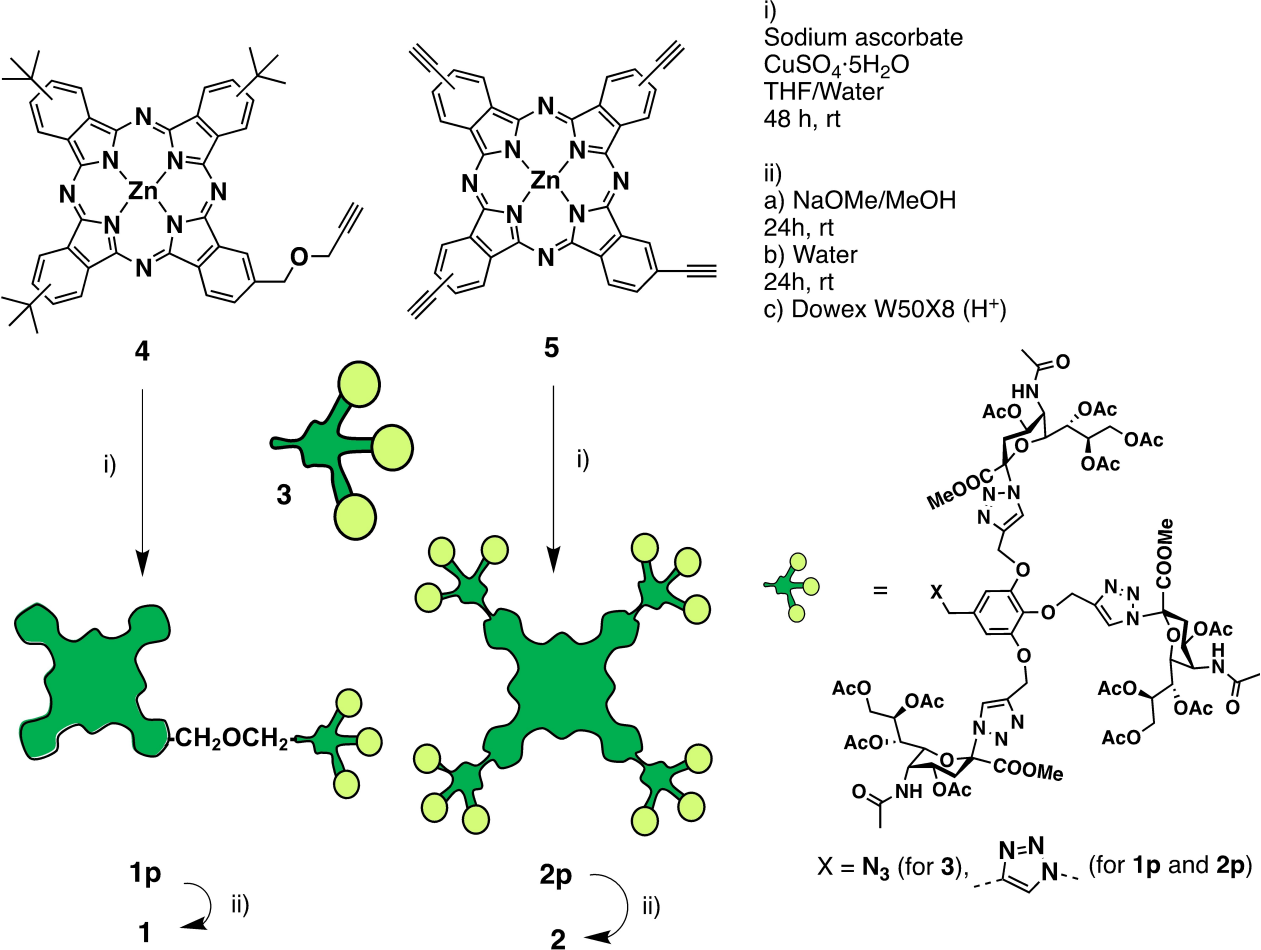
Synthetic route to the ZnPc‐SA dendritic compounds **1** and **2**, for which the detailed molecular structure is depicted in Figure 1.

The strategy to decorate ZnPc with multiple copies of SA is shown in Scheme 1 and, given the high molecular weight and functionality of the resulting hybrids, it was very challenging from the synthetic point of view. First, the glycodendron **3**, bearing three SA moieties (with the carboxylic and alcohol functionalities protected as methyl ester and acetylated groups, respectively), was prepared by copper‐catalyzed Huisgen cycloaddition (click) reaction between 3,4,5‐tris(propargyloxy)benzyl chloride **S1** and the azide‐containing derivative **S2**, followed by nucleophilic displacement of the chlorine atom with sodium azide (Scheme S1 in the Supporting Information). Compound **3** was then confronted in a second click reaction with either the asymmetrically substituted propargyloxy‐ZnPc **4** (prepared as in Scheme S2) or the symmetrically substituted tetraethynyl‐ZnPc **5** (prepared as in Scheme S3), leading to the corresponding dendritic ZnPc with protected SA units (**1 p** or **2 p**). Carbohydrate deprotection led to the final products **1** and **2**.

The click reactions of glycodendron **3** with ZnPc **4** or **5** were carried out with careful control of the stoichiometry (1.2 : 1 for the former and 4.8 : 1 for the latter), using CuSO_4_ ⋅ 5H_2_O as catalyst and sodium ascorbate as antioxidant agent, in mixtures of THF/water (see the Supporting Information for details). After 48 h, the reaction crudes were treated with QuadraSil MP metal scavenger resin to remove the excess of copper ions. The resulting products were purified by size exclusion chromatography (SEC) using Biobeads as stationary phase, obtaining **1 p** and **2 p** in 31 and 26 %, respectively. A deprotection step was then carried out by treatment with sodium methoxide in methanol (to remove the acetate groups), followed by addition of water to hydrolyze the SA methyl ester functionalities.^[35]^ The ZnPc‐SA biohybrids **1** and **2** were finally treated with Dowex W50X8 (H^+^) ion exchange resin, and triturated with acetone to eliminate the excess of acetic acid generated in the deprotection process, obtaining in quantitative yield two blue/green solids that were highly soluble in water.

The characterization of compounds **1 p** and **2 p** was performed by ^1^H NMR, electrospray ionization mass spectrometry (ESI‐MS) and UV/vis spectroscopy (see the Supporting Information). Their ^1^H NMR spectra showed clear correlation of integrals between the aromatic signals associated to the ZnPc core and those arising from the glycodendron protons (Figure S1). ESI‐MS allowed identifying peaks corresponding to the molecular ion of both compounds in various charged states (Figure S2). For the two final products, **1** and **2**, it was not possible to obtain a well resolved ^1^H NMR spectrum in D_2_O (and they are not soluble in sufficient concentrations in [D_6_]DMSO or [D_7_]DMF), due to their strong aggregation (especially in the case of **2**), which flattens and broadens all the signals, and to the presence of regioisomers in the case of **1** (Figures S3 and S5). ]The full deprotection of **1** and **2** was however demonstrated by the absence of peaks corresponding to the acetyl and methyl ester protecting groups in such ^1^H NMR spectra (Figure S3), while confirmation of the identity of **1** and **2** (with molecular masses of 2112.47 and 5866.62, respectively) came from ESI‐MS (Figure S4). Interestingly, high fragmentation was observed in the MS spectra, as expected from analysis of other glycodendron molecules reported in the literature.[^36^, ^37^] Finally, the purity of both compounds was proven by HPLC, confirming the great difference in polarity between **1** and **2** (Figure S5 and comments therein).

## Photochemical characterization

**Figure 2:**
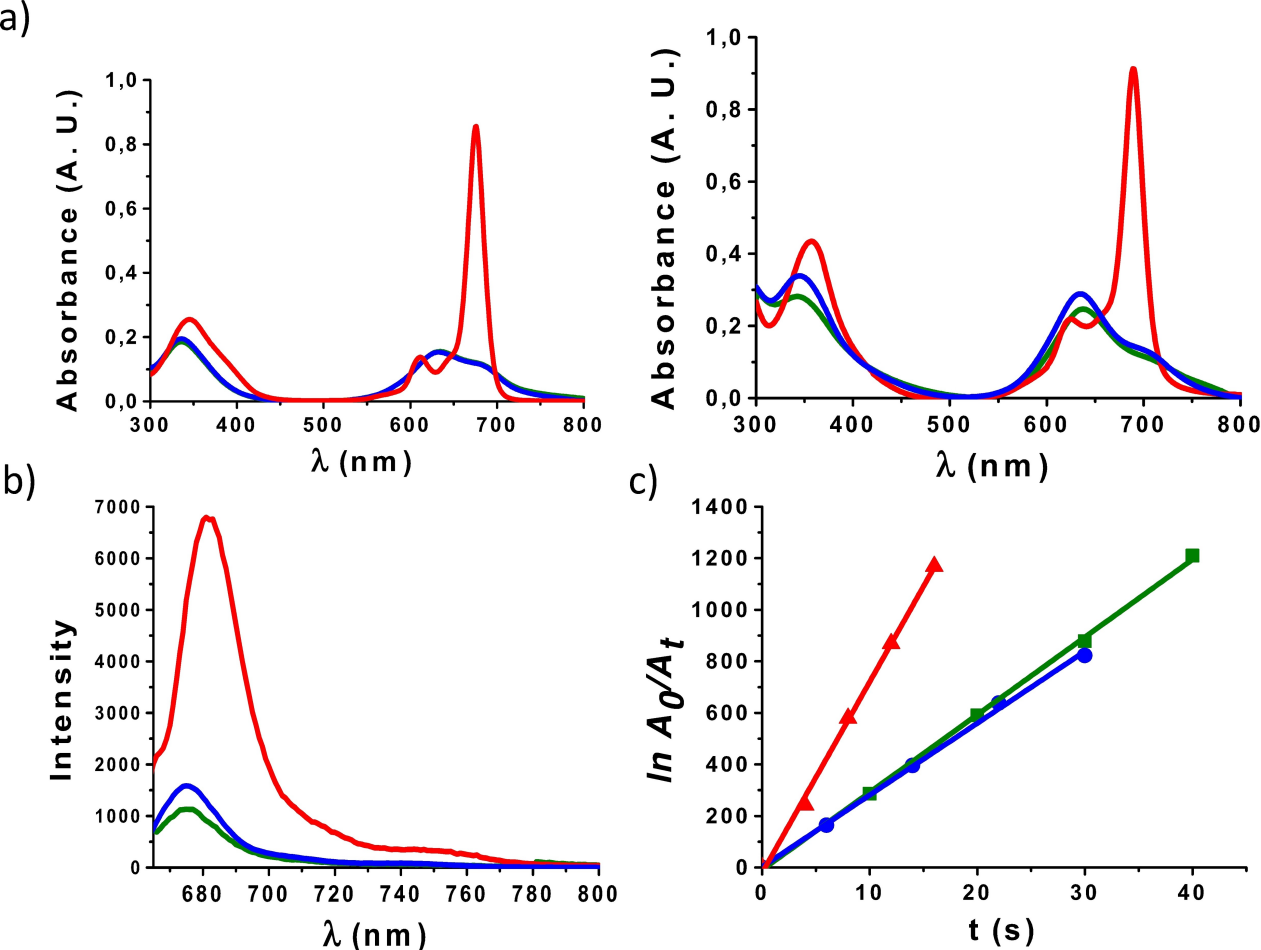
a) UV/vis spectra of **1** (left) and **2** (right) in DMF (red), water (green) and PBS (blue) at 7 μM. b) Fluorescence spectra of **1** (blue), **2** (green) and non‐substituted ZnPc (red) in DMF at 1 μM; *λ*_ex_=665, 679, and 660 nm, respectively. c) Plot of the decrease in DPBF absorption with time, photoinduced by **1** (blue), **2** (green) and non‐substituted ZnPc (reference compound, red), which correlates with the amount of singlet oxygen produced by each of these PS.

The UV/vis spectra of compounds **1** and **2** were recorded in different solvents: DMF, water and phosphate buffered‐saline (PBS) solution (Figure 2a). None of the compounds was aggregated in DMF, their spectra presenting the typical sharp Q‐band of monomeric ZnPc, with absorption maxima at 675 nm and 689 nm, respectively. In water or PBS, their absorbance decreased drastically and an intense hypsochromically displaced absorption showed up in the range of 630–640 nm. This behavior is indicative of H‐aggregation, and so an in‐depth comparative study of the self‐assembly of **1** and **2** is shown two sections below.

The photosensitizing capacity of the two ZnPc‐SA dendritic derivatives was evaluated in the non‐aggregating solvent DMF. Figure 2b shows the emission spectra of **1** and **2**, in comparison to that of non‐substituted ZnPc as reference. Irradiation was performed in all cases at a wavelength 10 nm lower than their absorption maxima, that is at 665 (for **1**), 679 (for **2**) and 660 nm (for non‐substituted ZnPc). The fluorescence quantum yield (*Φ*_F_) values of **1** and **2** were also determined to be 0.1 and 0.12, respectively (see section 1.3 in the Supporting Information). Singlet oxygen quantum yields (*Φ*_Δ_), in turn, were determined through the *relative method*,^[38]^ based on measuring the rate of photodegradation of a chemical scavenger (1,3‐diphenylisobenzofuran – DPBF) that is directly proportional to the formation of ^1^O_2_. Figure S6 shows the decay of the scavenger absorption induced by each of these two PS during different irradiation intervals. Decrease in Q‐band intensity or appearance of new bands were not observed, ensuring the PS integrity over the experiment. Under these conditions, plotting the dependence of ln(*A*_0_/*A*_t_) with irradiation time (*t*; with *A*_0_ and *A*_t_ being the DPBF absorbance values at 414 nm before and after the irradiation time *t*) afforded a straight line whose slope reflects the PS efficacy to generate ^1^O_2_ (Figure 2c), and from which *Φ*_Δ_ values of 0.46 and 0.48 could be calculated for **1** and **2**, respectively (Section 1.3 in the Supporting Information). These *Φ*_Δ_ values are slightly lower than that of the non‐susbtituted ZnPc reference (*Φ*_Δ (DMF)_=0.56),^[39]^ probably due to partial quenching of the excited state by the glycodendron units, yet they are good enough to permit an efficient photosensitizing action.

## Subcellular localization and phototoxicity experiments

**Figure 3:**
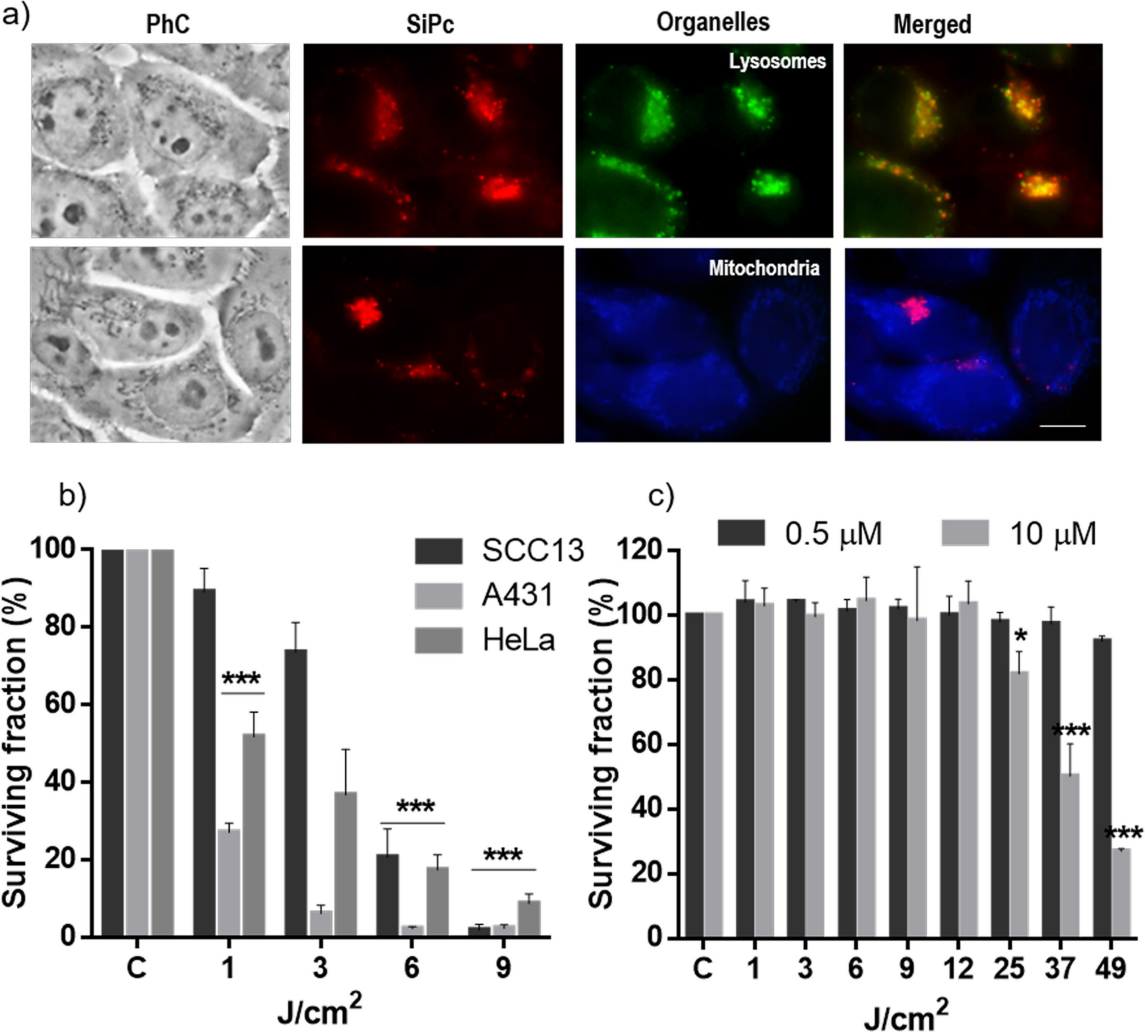
a) Subcellular localization of ZnPc‐SA **1** in SCC‐13 cells after 18 h of incubation at 10 μM of PS (similar data are presented for hybrid **2** in Figure S7); PhC: phase contrast. Red fluorescence is from the ZnPc, green fluorescence from lysosomes, and blue fluorescence from mitochondria. The merged image shows the ZnPC‐SA and each organelle together. A blue (450–490 nm) exciting lamp was used for LysoTracker (top row) and UVA (360–370 nm) exciting lamp was used for MitoTracker (bottom row), while green (545 nm) exciting light was used for the ZnPc‐SA derivative. Scale bar: 10 μm. b) Survival of SCC‐13, A431 and HeLa cells after 5 h of incubation with compound **1** (0.5 μM) followed by different light doses. c) Survival of SCC‐13 cells after 5 h of incubation with compound **2** (0.5 and 10 μM) followed by different light doses. Cell survival was evaluated 24 h after irradiation by the MTT assay. Each point corresponds to the mean value±SD obtained from three independent experiments. *P<0.05, **P<0.01, ***P<0.001.

In order to get an initial assessment of the therapeutic potential of PS **1** and **2**, we tested their capacity to enter into a representative cell line of human skin cancer, that is, SCC‐13. Despite of the strong aggregation of these compounds in water and PBS, we hypothesized that the presence of multiple copies of SA over the external surface of their assemblies could be beneficial for penetration across cellular membranes.^[40]^ Subcellular localization of **1** and **2** within SCC‐13 cells was evaluated by fluorescence microscopy, performing colocalization studies with fluorescent markers for specific organelles. To this end, cells were incubated with each dendritic ZnPc‐SA at a concentration of 10 μM, and then briefly washed with PBS. Assuming that no intracellular relocalization occurs, and in order to have a better fluorescence signal, the incubation time in this case was of 18 h, even if phototoxicity was measured after 5 h of incubation. The PS subcellular localization was anyway the same after 5 or 18 h incubation, the differences between both conditions being just in fluorescence intensity. The intracellular emission of both PS was coincident with that of the lysosomes, since a yellowish fluorescence was observed by overlapping of red and green emissions from the ZnPc and LysoTracker®, respectively (Figure 3a and S7a,b). Moreover, the fluorescence intensity was at the same level after incubation of the cells with both PS derivatives **1** and **2**, as monitored for more than 100 SCC‐13 cells using the J‐image program (Figure S7c). These results clearly point to an efficient and similar internalization of the two biohybrid PS.

Next, the phototoxicity of PS **1** and **2** upon red light irradiation was evaluated by the 3‐(4,5‐dimethylthiazol‐2‐yl)‐2,5‐diphenyltetrazolium bromide (MTT) assay. Dark toxicity was first discarded by incubation of the cells for 5 h with two different concentrations of both compounds (0.5 and 10 μM, as the minimum and maximum concentrations employed in the subsequent photosensitization experiments) in absence of light, showing no significant cytotoxicity, with survival rates above 95 % (Table S1). For PDT treatment, cells were incubated for 5 h with the lower non‐cytotoxic PS concentration (0.5 μM) and then exposed to different red light doses (1, 3, 6 and 9 J/cm^2^, *λ*=635 nm). MTT evaluation 24 h after the treatment revealed a drastic decrease of cell survival induced by PS **1** with a light dose of 9 J/cm^2^ in the three cell lines (Figure 3b). The extent of this decrease in cell survival after PS **1** photosensitization is completely comparable to that induced by other effective PS (e. g., LDH‐ZnPcS8 or silicaCe6‐FA), and also to PS precursors such as methyl aminolevulinate, which is used in clinic for several treatments of *in situ* SCC and pre‐malignant situations.[^41^, ^42^, ^43^]

In contrast, PS **2** did not induce any phototoxicity at those conditions (Figure 3c, dark gray bars), and both its concentration and the light dose had to be increased (up to 10 μM and 49 J/cm^2^, respectively) to observe a significant decrease in cell survival (Figure 3c, light gray bars). This remarkable difference in photodynamic performance, with the asymmetrically substituted compound **1** clearly outperforming the symmetric ZnPc‐SA compound **2**, could be related to the amphiphilic nature of the former, which let it get inserted in a disaggregated state in the membrane of lysosomes where it localizes within cells. We provide further support to this hypothesis in the next section.

In view of the promising photodynamic activity of hybrid **1**, an in‐depth in vitro study of this compound was performed. Besides SCC‐13, the possible applicability of PS **1** to human superficial cancers was demonstrated with a complete set of internalization, phototoxicity and cell morphology experiments with two other cell lines: HeLa and A431, associated to cervix and vulva tumors, respectively. In fact, one of the main applications of PDT is in non‐melanoma skin cancer, where PS or precursors are topically applied followed by light irradiation.^[43]^ In this direction, the subcellular localization of **1** in HeLa and A431 cells was also found to be in the lysosomes (Figure S8), while these two cell lines resulted even less resistant to the PDT treatment with **1** than SCC‐13 (Figure 3b, dark and light gray bars). The cell morphology of the treated cells was analyzed 24 h after the treatment using phase contrast microscopy, revealing for the three cell lines morphological changes that correlated with the irradiation time (Figure S9). The ratio of cells showing cytoplasmic retraction, with a rounded aspect similar to cells in apoptosis, gradually increased with the light dose applied. Although their visualization was difficult because dead cells get easily detached from the plate, they represented the majority for those irradiated with 9 J/cm^2^. Such images are in concordance with the results obtained from cell viability assays.

## Aggregation studies and intracellular ROS generation

The huge difference in the photodynamic performance of compounds **1** and **2** made us reflect about the reason for such dissimilar behavior. It is common that anionic and hydrophilic aggregated drugs (like **1** and **2**) enter the cell through endocytosis, and so are localized in lysosomes.^[44]^ That explains that both PS **1** and **2** go to the lysosome and do not stay at the plasma membrane. However, that aggregated state normally results in a lower photodynamic efficacy shown by lysosomal localization, versus compounds that locate in other organelles, due to the excitonic coupling that occurs when a PS aggregates through π‐π stacking promoted by the aqueous medium. This is what happens with the ZnPc‐SA biohybrid **2**, as Figure 2a (right) reveals a strong aggregation by π‐π stacking in water and PBS, and the measured octanol/water partition coefficient (log *P*_OW_=−2.23; Section 1.4 in the Supporting Information and Figure S10) confirms the very hydrophilic nature of those aggregates.

The ZnPc‐SA hybrid **1**, in contrast, performs much more efficiently in the PDT experiments, which could be related to its amphiphilic structure, with three isoindole units substituted with *tert*‐butyl groups and the fourth one substituted with a very hydrophilic SA‐containing dendron. Indeed, Figure 2a (left) shows a lower tendency to aggregate (the band at 630–640 nm is significantly less prominent), and its log *P*_OW_ value of −1.52 (Figure S10) indicates a lower preference for the aqueous phase. Reverse phase HPLC measurements in a 200‐C18‐42 (ACE 3C18‐AR, 150×3 mm, 3 μm) column, using a water/acetonitrile gradient as mobile phases and a flow rate of 0.5 mL/min, confirmed a great difference in polarity between **2** (*t*_R_=0.92 min in water/acetonitrile 60 : 40) and **1** (*t*_R_>6.4 min in 100 % acetonitrile; Figure S5), with the advantage that HPLC in such conditions reflects the compounds polarity in their monomeric state. Our hypothesis was then that PS **1** could get inserted into the membrane of lysosomes in its monomeric form,^[45]^ with the macrocyclic core embedded in the hydrophobic region of the lipid bilayer and the hydrophilic dendron exposed to the aqueous environment on the membrane surface. To prove this hypothesis, we carried out a comparative study to correlate the aggregation behavior of hybrids **1** and **2** with their capacity for intracellular ROS generation.

**Figure 4:**
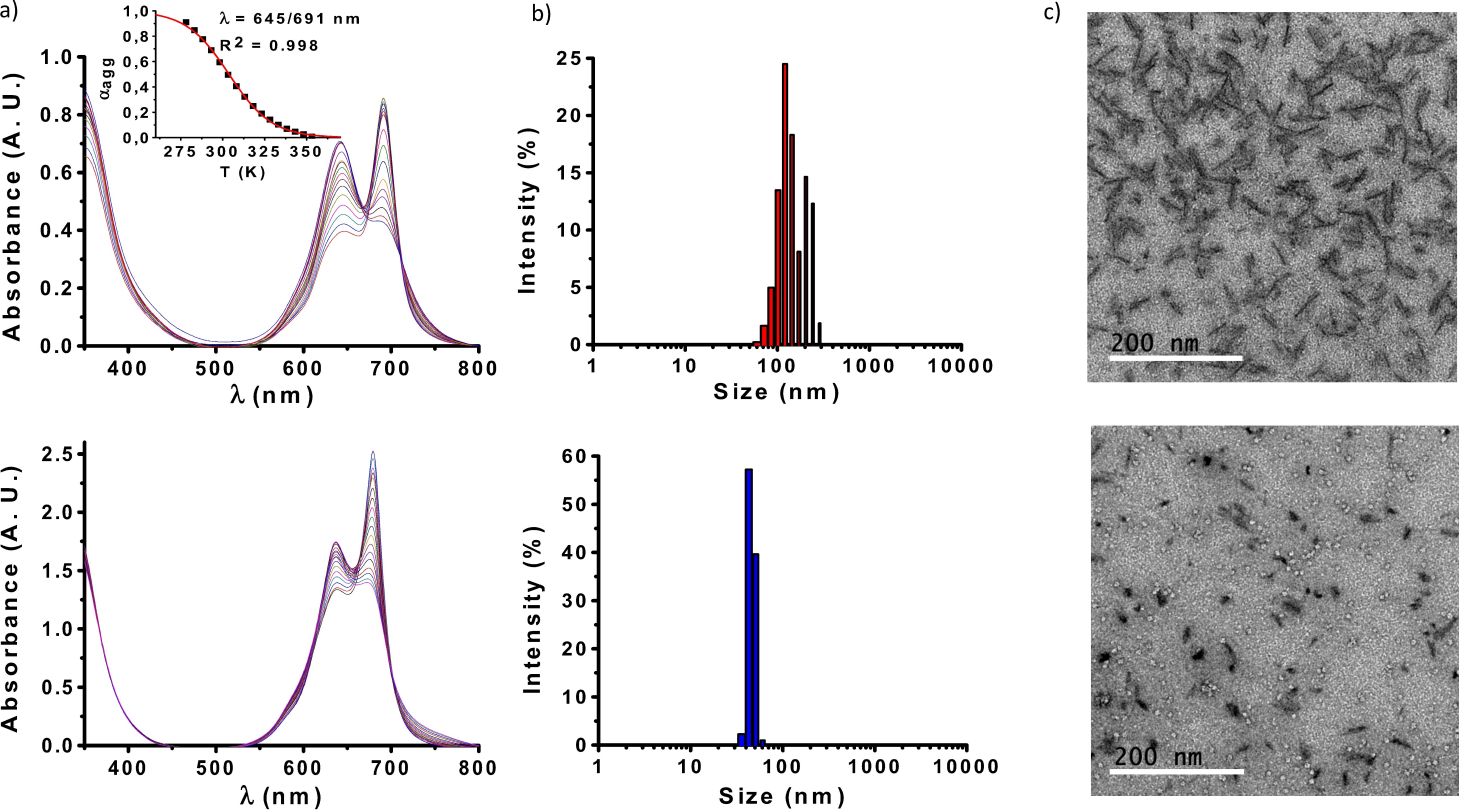
a) Cooling curves of 50 μM **1** in DMF/PBS (30 : 70; bottom) and 15 μM **2** in DMF/PBS (35 : 65; top) over temperature ranges of 90 to 0 °C and 80 to 5 °C, respectively, with temperature decrease steps of 5 °C. Inset: Fitting of the cooling curve of **2** to an isodesmic supramolecular polymerization model. b) DLS size distribution diagrams of 5 μM **1** (bottom) and **2** (top) in PBS. c) TEM micrographs of 50 μM **1** (bottom) and **2** (top).

The supramolecular organization of hybrids **1** and **2** was first examined through temperature‐dependent UV/vis experiments. Aggregation in PBS was too strong to be disrupted by heat, and so solvent mixtures with different proportions of PBS and DMF were tested to study the aggregation mechanism. Figure 4a, top panel, shows for example the absorption spectra of compound **2** in DMF/PBS (35 : 65), recorded after successive temperature decrease steps of 5 °C in the range from 80 to 5 °C, revealing two isosbestic points at 670 and 712 nm. The degree of aggregation (*α*_agg_) could be calculated from that set of spectra by a standard method (see Section 1.5 in Supporting Information). Plotting *α*_agg_ as a function of temperature gave rise to a sigmoidal curve (Figure 4a top, inset), which is indicative of an isodesmic supramolecular polymerization mechanism.[^46^, ^47^, ^48^, ^49^] Analysis of the data following an isodesmic model resulted in a K_α_ value of 2.39×10^5^ M^−1^, while the van't Hoff plot obtained from such analysis allowed determining the rest of thermodynamic parameters of the assembly process (Figure S11 and Table S2).

For the case of compound **1**, cooling curves were recorded at different proportions of DMF/PBS (ranging from 10 to 40 % DMF), but it was not possible to extract the supramolecular thermodynamic parameters, as the obtained *α*_agg_ data did not fit into a sigmoidal curve for any of those solvent ratios. Along the series it could be observed, however, that the aggregation strength drastically decreased with the amount of DMF. Disruption of aggregates by heat was almost negligible in DMF/PBS 10 : 90 (Figure S12a), while in DMF/PBS 40 : 60, aggregation was almost inexistent at room temperature (Figure S12c). Finally, at a solvent mixture (DMF/PBS 30 : 70) comparable to that in which the optimum cooling curve was obtained for compound **2**, it was possible to discern that the aggregation of **1** (Figure 4a bottom panel) is less prominent than that of hybrid **2** (top panel).

As the self‐assembly features of PS can determine their photodynamic response, the size and morphology of the aggregates formed by **1** and **2** were also characterized. Their hydrodynamic diameter was estimated by dynamic light scattering (DLS), carried out in PBS at a low concentration of PS (5 μM) in order to minimize the ZnPc absorption (Figure 4b). Compound **2** yields large and relatively polydisperse assemblies with an average diameter of 150±76 nm (Figure 4b, top). Compound **1**, in turn, yields rather monodisperse, smaller assemblies with an average diameter of 48±11 nm (Figure 4b, bottom). Transmission electron microscopy (TEM) images from 50 μM PS samples deposited on hydrophilic glow‐discharged carbon‐coated copper grids confirmed those observations (Figures 4c and S13). Such a high concentration was used to ensure the presence of sufficient, visible amounts of objects on the grids coming from the sample, assuming that the same type of nanoaggregates would be present at the lower concentrations employed for PDT. While rods with a well‐defined diameter of 8±3 nm and lengths in the range of 40–50 nm were widespread over the grid for compound **2** (Figure 4c, top panel), the aggregation of **1** resulted in a majority of irregular, spherical assemblies with diameters in the range of 10–20 nm (Figure 4c, bottom panel). Importantly, the divergence in sizes observed by DLS and TEM analysis (larger for the former) is a common phenomenon in studies of nanostructures, and relates to the fact that DLS determines diffusion coefficients, from which average particle sizes can be derived, yet various factors can weight differently the resulting particle size distributions.[^50^, ^51^]

**Figure 5:**
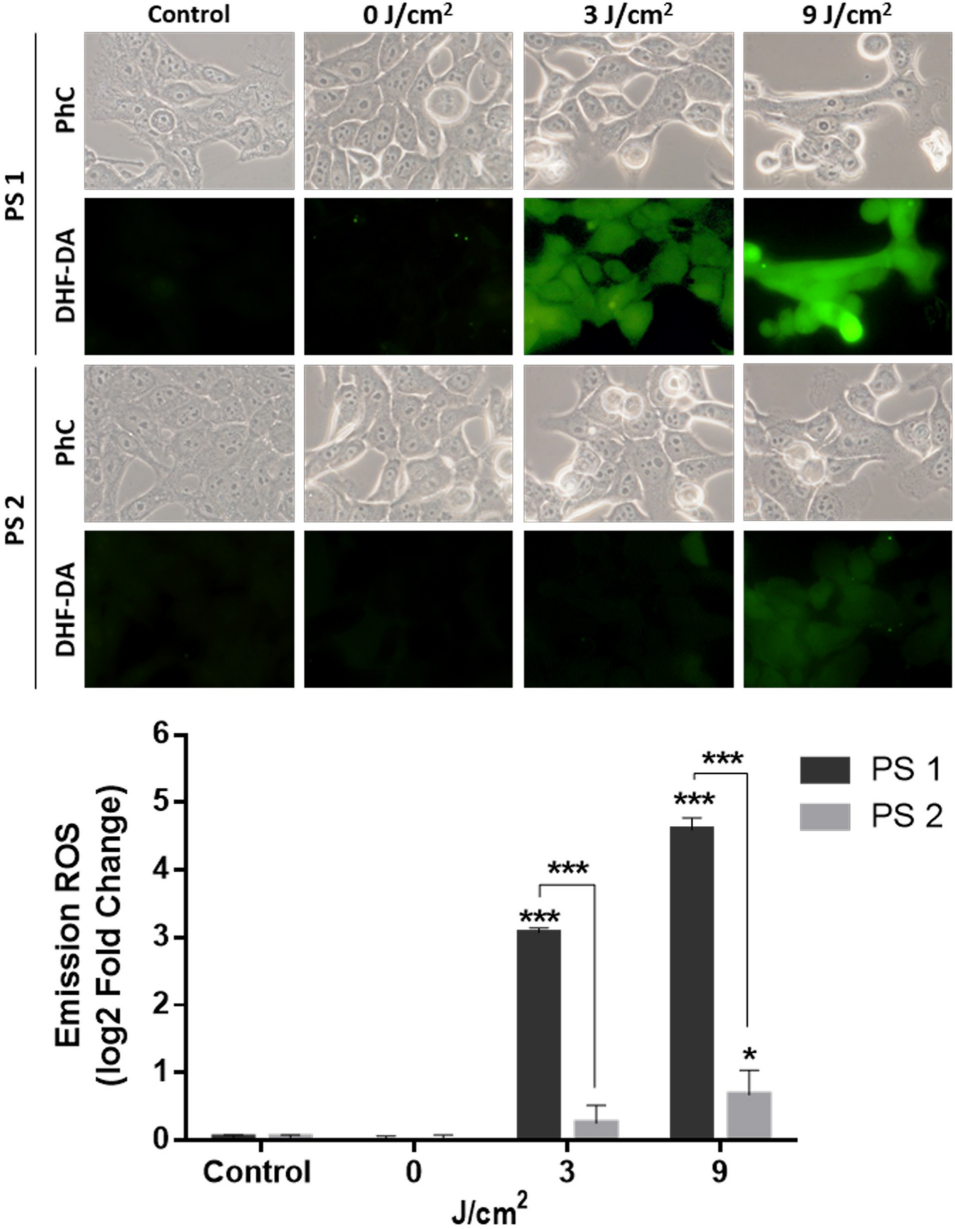
ROS production detected by the DHF‐DA fluorescent probe after PDT with the ZnPc‐SA biohybrids **1** (top) and **2** (bottom) under irradiation with different red light doses. 1st column: Control experiment in the absence of PS. 2nd column: Cells maintained in the dark, as a second control. 3rd and 4th columns: Cells irradiated with two different light doses. A fluorescence signal indicative of ROS production was observed by fluorescence microscopy (*λ*_ex_=436 nm). The intracellular fluorescence intensity was measured by ImageJ (B). *P<0.05, ***P<0.001. PhC: phase contrast images, for analysis of the effect of the PDT treatment on the cells morphology.

The above results provide useful insights about which could be the behavior of **1** and **2** in the intracellular medium. Compound **2** self‐assembles into larger structures that cannot be easily disrupted. The amphiphilic PS **1**, on the contrary, tends to form smaller assemblies, which are less robust and could be disassembled in the presence of a membrane, resulting in monomeric PS molecules with no exciton coupling quenching the excited state. To check if that is the case when hybrid **1** reaches the lysosomes in the studied target cells (see above), we analyzed the intracellular ROS formation in SCC‐13 cells by fluorescence microscopy, when subjected to PDT with PS **1** and **2**, using the 2,7‐dichloro‐dihydrofluorescein diacetate (DHF‐DA) fluorescent probe (Figure 5).

SCC‐13 cells were incubated with 0.5 μM **1** or **2** for 4 h, and 6 μM DHF‐DA was then added and incubated for 1 additional h. Immediately after exposure to red light (*λ*=635 nm, 3 or 9 J/cm^2^), the cell cultures were analyzed by fluorescence microscopy, under blue light excitation (*λ*_ex_=436 nm). As shown in Figure 5, the control samples, consisting of cells in absence of PS (1st column images) or non‐irradiated (2nd column images), exhibited a negligible green fluorescent signal. The experiment with PS **1** under light illumination showed, on the other hand, a clear light dose‐dependent increase in fluorescence (compared to baseline levels, see the inset graph), revealing prominent ROS production after PDT. Comparison with the response of PS **2** actually reveals a substantially higher ROS photoproduction by the ZnPc‐SA biohybrid **1**, which confirms its in‐situ intracellular activation when this amphiphilic compound gets inserted into the lysosomal membrane.

## Conclusions

Here, we have designed and synthesized a novel dendritic biohybrid PS with structural features and self‐assembly properties that are suitable to address key aspects of photodynamic therapy in the intracellular medium. A challenging synthetic strategy allowed us decorating ZnPc derivatives with multiple copies of SA, a carbohydrate that confers high water‐solubility, biocompatibility, internalization capacity, and enhanced PS transport. Self‐assembly of the resulting biohybrids, either in aqueous solution or in the membrane of target cellular organelles (lysosomes), seems to depend on the number and distribution of SA moieties over the ZnPc macrocycle. This has led to an insightful inference about the interplay between self‐assembly and photochemical activity in the engagement of PS transport, cellular internalization and on‐site activation, which overall are responsible for triggering an efficient photodynamic response against different superficial cancer cell lines.

## Conflict of interest

The authors declare no conflict of interest.

## Supporting information

As a service to our authors and readers, this journal provides supporting information supplied by the authors. Such materials are peer reviewed and may be re‐organized for online delivery, but are not copy‐edited or typeset. Technical support issues arising from supporting information (other than missing files) should be addressed to the authors.

Supplementary
