# Supplementary material for: Tuning the Nanoaggregates of Sialylated Biohybrid Photosensitizers for Intracellular Activation of the Photodynamic Response
Source: Chemistry. 2021 May 17;27(37):9634–42. doi: 10.1002/chem.202100681 (PMC8360122; doi:10.1002/chem.202100681)
Supplement: Supplementary file 1 — Supplementary [file CHEM-27-9634-s001.pdf]

Chemistry–A European Journal

Supporting Information

Tuning the Nanoaggregates of Sialylated Biohybrid Photosensitizers for Intracellular Activation of the Photodynamic Response

Verónica Almeida-Marrero, Marta Mascaraque, María Jesús Vicente-Arana, Ángeles Juarranz,* Tomás Torres,* and Andrés de la Escosura*

Table of Contents

1. **Materials and Methods**

   1.1. Synthesis of SA-ZnPc biohybrids.

   1.2. Characterization of SA-ZnPc biohybrids.

   1.3. Fluorescence and singlet oxygen measurements.

   1.4. Determination of partition coefficient in octanol/water.

   1.5. Supramolecular polymerization studies.

   1.6. Subcellular localization and phototoxicity studies.

2. **Figures and Tables**

   Figure S1: ^{1}H-NMR spectra of **1p** and **2p**.

   Figure S2: ESI mass spectra of **1p** and **2p**.

   Figure S3: Comparison of ^{1}H-NMR spectra of **1p** and **1**, and **2p** and **2**.

   Figure S4: ESI mass spectra of **1** and **2**.

   Figure S5: HPLC chromatograms of **1** and **2.**

   Figure S6: Singlet oxygen experiments.

   Figure S7: Subcellular localization of compound **2** in SCC-13 cells.

   Table S1: Dark toxicity data of **1** and **2** in the different cell lines.

   Figure S8: Subcellular localization of compound **1** in A431 and HeLa cells.

   Figure S9: Cell morphology studies.

   Figure S10: Partition coefficient UV-Vis experiments for **1** and **2** in octanol/water.

   Figure S11: Van't Hoff analysis of the supramolecular polymerization of **2**.

   Table S2: Thermodynamic parameters of the self-assembly of compound **2**.

   Figure S12: UV-Vis temperature-dependent aggregation studies of **1**.

   Figure S13: TEM micrographs of the assemblies formed by **1** and **2**.

1. Materials and Methods

Inert conditions under argon atmosphere and standard glassware were used to perform all reactions. To monitor them, TLC plates pre-coated with silica gel 60-F254 (Merck) were used. Column chromatography was carried out on Merck silica gel 60, 40-63 μm (230-400 mesh), and Biorad Biobeads SX-1 (200-400 mesh) were used as stationary phase for Size Exclusion Chromatography (SEC). ^{1}H and ^{13}C-NMR spectra were performed using Bruker DRX 500, Bruker Avance and Bruker Avance II (^{1}H: 300 MHz and 500 MHz, ^{13}C: 75 MHz and 125 MHz) spectrometers. The internal references of all spectra were made using the residual solvent (^{1}H: δ = 7.26 for $CDCl_3$, δ = 2.49 for DMSO-$_{d6}$) or solvent resonances (^{13}C: δ = 77.0 for $CDCl_3$) relative to $SiMe_4$, respectively. JASCO V-660 and JASCO FP-8600 spectrophotometers were used to measure UV-Vis and fluorescence, respectively. IR spectra (IR) were performed on a FT-IR Cary 630 (Agilent Technologies) using ATR as technique, correcting the intensity by ATR algorithm. Matrix-assisted laser desorption/ionization time of flight (MALDI-TOF) was recorded using a Bruker Ultrareflex III spectrometer. Electrospray ionization source (ESI) in positive or negative mode was performed using an ultra-high-resolution QTOF instrument (MAXIS II, Bruker). HPLC chromatograms were recorded using a HPLC Agilent 1100 Series system and 200-C18-42 (ACE 3 C18-AR, 150x3mm, 3μm) column. Mass and HPLC experiments were performed at the Servicio Interdepartamental de Investigación (SIdI) of the Universidad Autónoma de Madrid. The size of aggregates was measured using Dynamic Light Scattering (DLS) with a Nanotrac Wave particle size analyser. The obtained data are expressed as an average of 3 measurements at 22 ºC. TEM images were recorded using a JEOL JEM 1400 transmission electron microscope working at 100 kV, at the Centro Nacional de Microscopía of the Universidad Complutense de Madrid. Carbon-coated copper grids were subjected to 1 cycle of glow discharge of 20 s to make them hydrophilic. Grids were covered with 20 μL of sample for 5 min, washed with water and 5 μL of uranyl acetate (1% w/v) was applied as staining for 45 s. After that, the excess was removed by a piece of filter paper.

1.1. Synthesis of SA-ZnPc biohybrids

1.1.1. ***Scheme S1.*** *Synthesis of glycodendron* ***3***

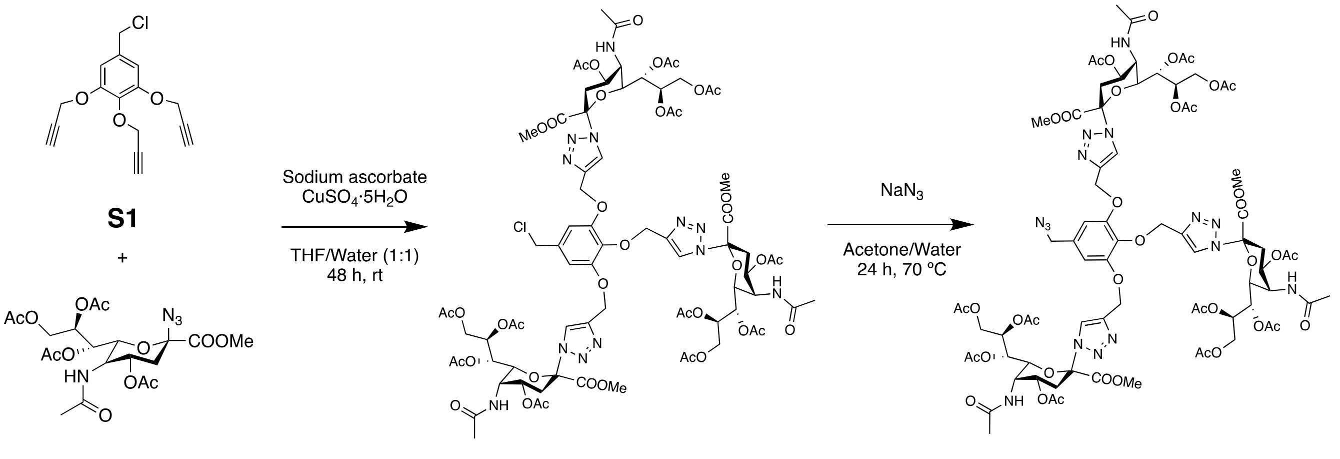

S2 **S3** **Glycodendron 3**

Compounds **S1**[1] and **S2**[2,3] have been prepared following described procedures.

- *Synthesis of compound **S3***

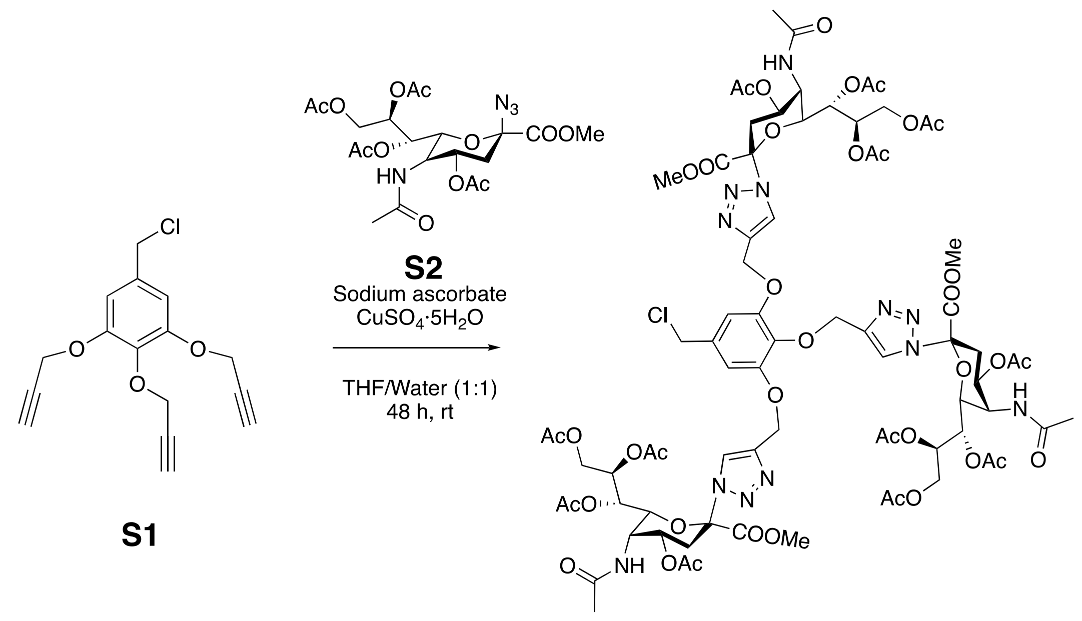

S3

S1[1] (0.41 g, 1.42 mmol), **S2**[2,3] (2.65 g, 5.13 mmol) and sodium ascorbate (0.25 g, 1.28 mmol) were placed into a two-necked round-bottom flask under argon atmosphere. THF (10 mL) and distilled water (10 mL) were added with a syringe, and $CuSO_4·5H_2O$ (0.32 g, 1.28 mmol) was added under argon atmosphere. The reaction mixture was stirred for 48 h at rt. After that, THF was evaporated, and the resulting mixture was diluted with DCM, extracted with water, dried over anhydrous $MgSO_4$, and filtered. The crude was then purified by column chromatography, using a gradient from EtOAc to EtOAc/MeOH (10%) as eluent. Yield: 88 % (2.30 g).

^{1}**H-NMR** (300 MHz, $CDCl_3$): δ (ppm) = 8.1 – 8.0 (m, 3H; triazole), 6.79 (s, 2H; H_{ar}), 5.7 – 5.6 (m, 3H; NH), 5.4 – 5.2 (m, 15H; $H7_{SA}$, $H8_{SA}$, $H4_{SA}$, O-C$\underline{H}_2$-triazole), 4.49 (s, 2H; Cl-C$\underline{H_2}$), 4.4 – 4.3 (m, 3H; $H9_{SA}$), 4.2 (m, 3H; $H6_{SA}$), 4.1 – 4.0 (m, 6H; $H9_{SA}$, $H5_{SA}$), 3.73 (s, 9H; COOMe), 3.4 – 3.3 (m, 3H; $H3_{SA,eq}$), 2.7 – 2.6 (m, 3H; $H3_{SA,ax}$), 2.1 –2.0 (m, 36H; CH_3-acetyl), 1.86 (s, 9H; CH_3CON).

^{13}**C-NMR** (125 MHz, $CDCl_3$): δ (ppm) = 170.813, 170.682, 170.618, 170.484, 170.413, 170.377, 170.189, 170.105, 170.041, 166.437, 166.418, 152.388, 144.739, 144.450, 144.297, 137.975, 133.531, 122.688, 122.549, 122.420, 109.448, 107.341, 88.610, 88.571, 88.534, 77.160, 73.827, 73.754, 68.635, 68.313, 68.256, 67.998, 67.039, 65.975, 64.840, 63.269, 62.356, 62.240, 54.181, 54.128, 49.413, 49.321, 46.361, 36.284, 36.191, 29.731, 23.223, 21.205, 20.879, 20.808, 20.788, 13.653.

FT-IR (film): ν (cm^{-1}) = 3483, 2964, 1754, 1673, 1598, 1557.

HR-MS (ESI Positive, MeOH + 0.1% formic acid): Calc for $C_{76}H_{97}ClN_{12}NaO_{39}$ $[M+Na]^+$: *m/z*: 1859.5557, found 1859.5488.

- *Synthesis of glycodendron **3***

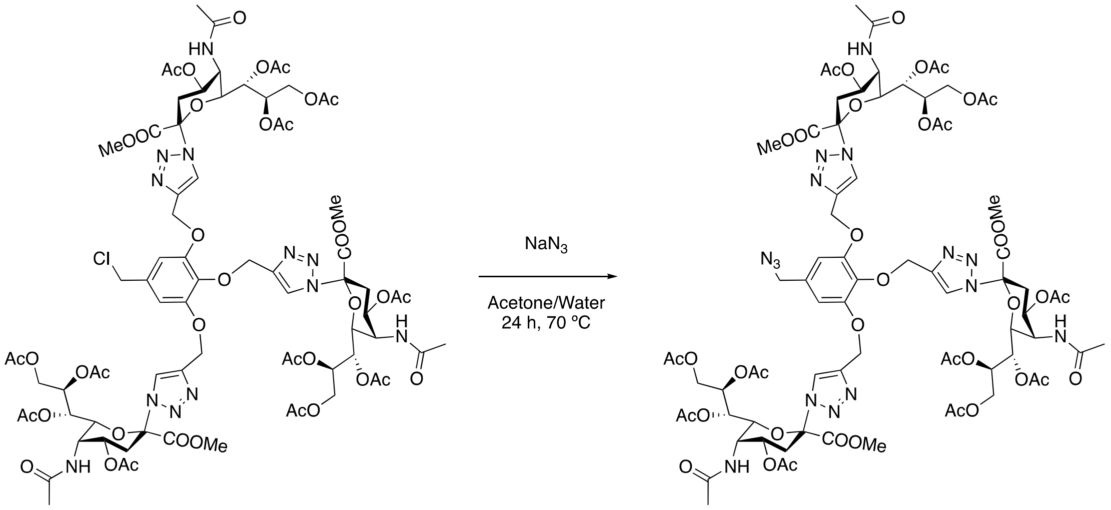

S3 **Glycodendron 3**

S3 (1.5 g, 0.82 mmol) and NaN_3 (0.08 g, 1.22 mmol) were dissolved in 20 mL of water/acetone (1:1), and the mixture was stirred for 24 h at 70ºC. Acetone was evaporated and the compound was extracted with DCM, washed with water and brine, dried over anhydrous $MgSO_4$, and filtered. The solvent was then vacuum evaporated, to afford the pure compound as a light yellow solid. Yield: 95% (1.5 g).

^{1}**H-RMN** (300 MHz, $CDCl_3$): *δ* (ppm) = 8.1(m, 3H; triazole), 6.76 (s, 2H; H_{ar}), 5.4 – 5.2 (m, 18 H; NH, $H7_{SA}$, $H8_{SA}$, $H4_{SA}$, O-C<u>H</u>$_2$-triazole), 4.4 – 4.2 (m, 8H; N_3-CH_2, $H9_{SA}$, $H6_{SA}$), 4.1 – 4.0 (m, 6H; $H9_{SA}$, $H5_{SA}$), 3.77 (s, 9H; COOMe), 3.40 (m, 3H; $H3_{SA,eq}$), 2.7 – 2.6 (m, 3H; $H3_{SA,ax}$), 2.2 – 2.0 (m, 36H; CH_3-acetyl), 1.91 (s, 9H; CH_3CON).

^{13}**C-NMR** (75 MHz, $CDCl_3$): *δ* (ppm) = 170.802, 170.744, 170.678, 170.617, 170.498, 170.432, 170.400, 170.195, 170.105, 170.051, 166.406, 152.573, 152.348, 144.752, 144.459, 144.278, 137.830, 131.662, 122.700, 122.523, 122.425, 108.938, 107.365, 88.627, 88.559, 77.160, 73.806, 73.734, 68.619, 68.501, 68.318, 68.255, 67.992, 67.049, 65.985, 63.287, 63.136, 62.335, 62.217, 54.707, 54.178, 54.099, 49.447, 49.348, 36.199, 23.944, 23.211, 21.186, 20.864, 20.768, 19.706, 13.612.

FT-IR (film): *ν* (cm^{-1}) = 3483, 2964, (N_3) 2106, 1754, 1673, 1598, 1557.

HR-MS (ESI Positive, MeOH): Calc for $C_{76}H_{97}N_{15}NaO_{39}$ $[M+Na]^+$: *m/z*: 1866.5960, found 1866.5981.

1.1.2. ***Scheme S2.*** *Synthesis of the ZnPc precursor* ***4***

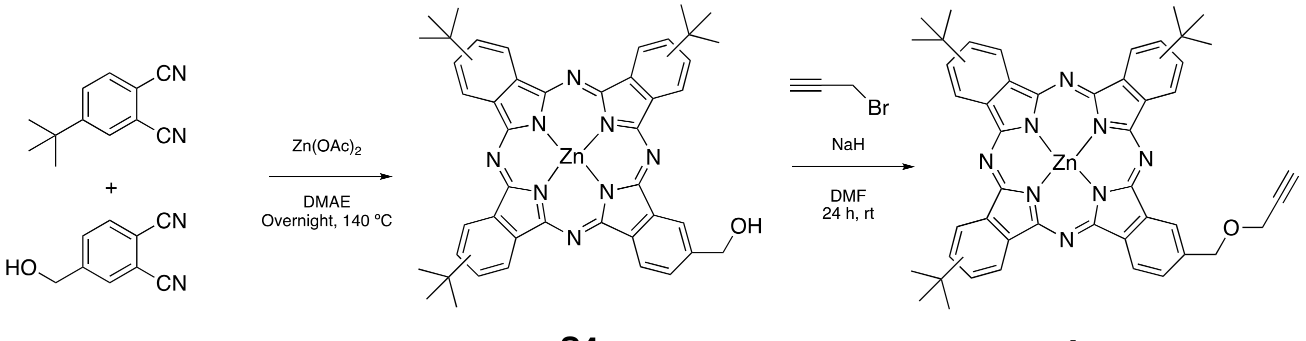

S4 **4**

4-hydroxymethylphthalonitrile and the hydroxymethyl-ZnPc **S4** have been prepared following described procedures.[4]

- *Synthesis of propargyloxy-ZnPc* ***4***

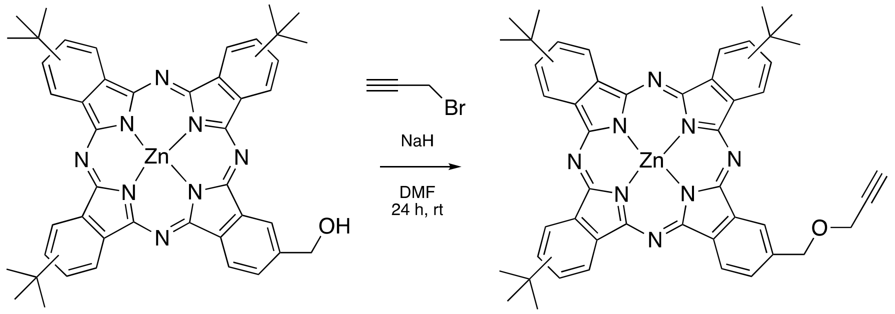

S4 **4**

S4[4] (0.12 g, 0.15 mmol) was dissolved in dry DMF (3 mL), NaH (0.007 g, 0.306 mmol) was added at 0 ºC, and the reaction was stirred for 1 h at that temperature. After that, propargyl bromide (0.036 g, 0.3 mmol) was added at 0ºC, and the reaction was stirred for 24 h at rt. The solvent was then evaporated, and the crude was extracted with DCM, washed with water, dried over anhydrous $MgSO_4$, and filtered. The solvent was evaporated under reduced pressure. The product was purified by column chromatography, using a mixture of heptane/dioxane (4:1) as eluent, obtaining a dark blue sticky solid. Yield: 80 % (0.1 g).

^{1}**H-NMR** (300 MHz, $DMSO_{d6}$): *δ* (ppm) = 9.4 – 9.1 (m, 8H; H_{ar}), 8.4 – 8.3 (m, 3H; H_{ar}), 8.2 – 8.1 (m, 1H; H_{ar}), 5.2 – 5.1 (m, 2H; CH_2-O), 4.6 – 4.5 (m, 2H; O-C<u>H</u>$_2$-CH), 3.7 (m, 1H; CH), 1.81 (s, 27H; CH_3).

FT-IR (film): *v* (cm^{-1}) = 3304, 2952, 2857, 1662, 1612, 1561.

HR-MS (MALDI-TOF, matrix DCTB): Calc for $C_{48}H_{44}N_8OZn$ $[M]^+$: *m/z*: 812.2924, found 812.2925.

UV-Vis (DMF): λ_{max} (nm) (log *ε)* = 687 (5.15), 645 (4.36), 611 (4.36), 352 (4.68).

1.1.3. ***Scheme S3.*** *Synthesis of the ZnPc precursor* ***5***

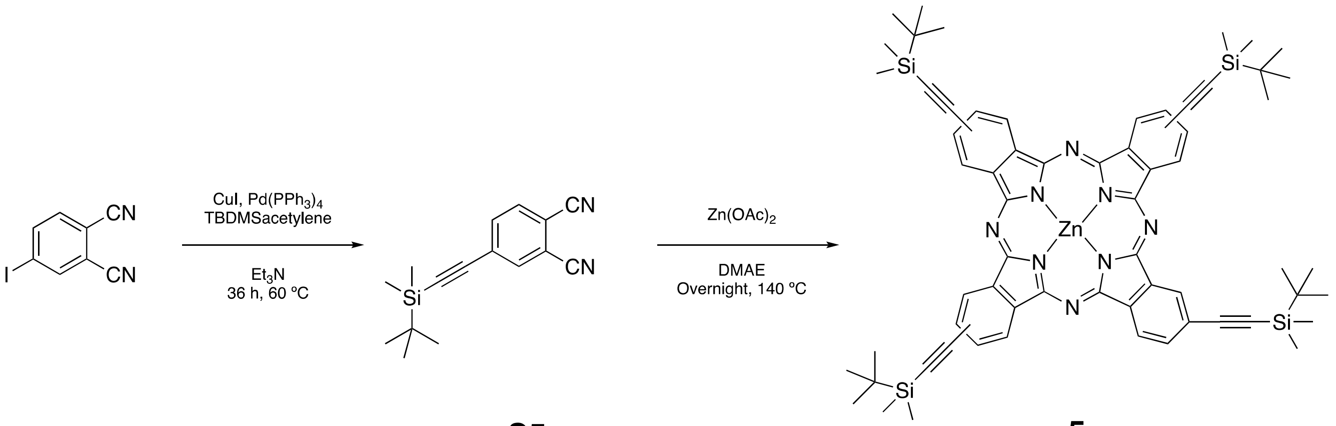

S5 **5**

4-iodophthalonitrile has been prepared following a described procedure.**[5]**

- *Synthesis of 4-((tert-butyldimethylsilyl)ethynyl)phthalonitrile (**S5**).*

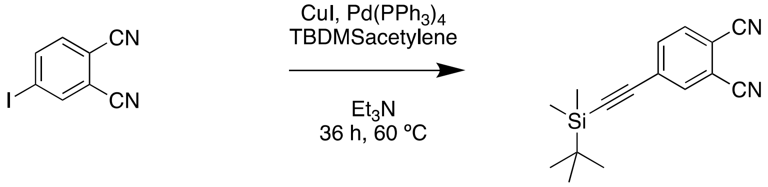

S5

4-iodophthalonitrile[5] (1.5 g, 5.9 mmol) and CuI (0.23 g, 1.18 mmol) were placed into a Schlenk tube, and the system was filled and evacuated 3 times with argon. Then, freshly distilled Et_3N (30 mL) was added with a syringe, and 3 cycles of freeze-pump-thaw were applied. After that, $Pd(PPh_3)_4$ (0.34 g, 2.95 mmol) and (tert-butyldimethylsilyl)acetylene (1.49 mL, 7.97 mmol) were added under argon atmosphere, and the reaction was stirred for 36 h at 60 ºC. The reaction was quenched using 10% aq. NH_3 and the crude product was extracted with $CHCl_3$. The organic layer was washed with 10% aq. NH_4Cl and dried over anhydrous $MgSO_4$. The solvent was evaporated, and the crude residue was purified by column chromatography, using DCM/heptane (1:1) as eluent, to afford a yellow solid. Yield: 80 % (1.25 g).

^{1}H-NMR (300 MHz, $CDCl_3$): *δ* (ppm) = 7.85 (t, *J* = 1.2 Hz, 1H; H6), 7.74 (d, *J* = 0.9 Hz, 2H; H1, H4), 0.99 (s, 9H; $(CH_3)_3$), 0.21 (s, 6H; CH_3).

^{13}C-NMR (75 MHz, $CDCl_3$): *δ* (ppm) = 136.57, 136.11, 133.52, 129.24, 116.38, 115.21, 114.71, 102.17, 101.48, 26.19, 16.81, -4.78.

FT-IR (film): *v* (cm^{-1}) = 2948, 2920, 2849, 2230, 1593.

HR-MS (EI Positive): Calc for $C_{16}H_{18}N_2Si$ $[M]^+$: *m/z*: 266.1239, found 266.1232.

- *Synthesis of symmetrically substituted tetraethynyl-ZnPc (**5**)*

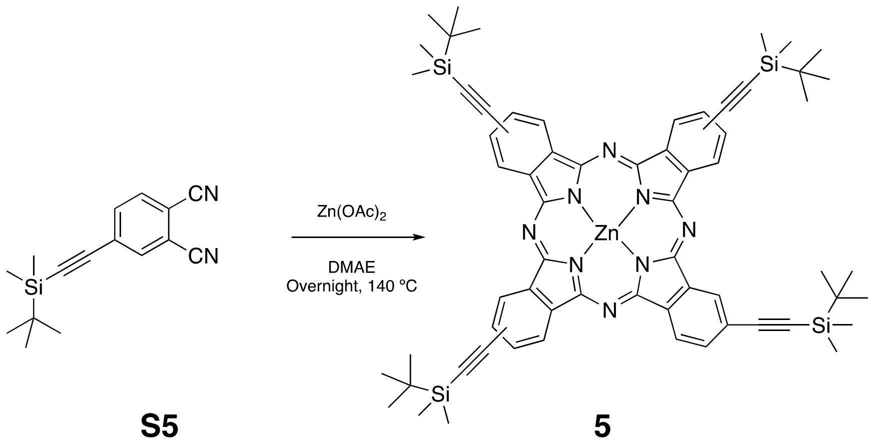

S5 **5**

4-((*tert*-butyldimethylsilyl)ethynyl)phthalonitrile (**S5**) (0.2 g, 0.75 mmol), $Zn(OAc)_2$ (0.37 g, 0.2 mmol) and DMAE (3 mL) were placed into a two-necked round-bottom flask, under argon atmosphere. The reaction was stirred overnight at 140 ºC. After that, the reaction was cooled down to room temperature and DMAE was vacuum evaporated. The solution was poured onto a mixture of water/metanol (4:1) and then filtered. The crude product was purified by column chromatography, using heptane/dioxane (4:1) as eluent, obtaining a dark green powder. Yield: 20 % (0.17 g).

^{1}H-NMR (300 MHz, THF_{d8}): δ (ppm) = 9.2 – 8.8 (m, 8H; H_{ar}), 8.2 – 8.1 (m, 4H; H_{ar}), 1.3 (m, 36H; $(CH_3)_3$), 0.51 (s, 24H; CH_3).

FT-IR (film): v (cm^{-1}) = 2946, 2924, 2853, 2143.

HR-MS (MALDI-TOF, matrix DCTB): Calc for $C_{64}H_{72}N_8Si_4Zn$ $[M]^+$: *m/z*: 1128.4243, found 1128.4250.

UV-Vis (DMF): λ_{max} (nm) (log ε) = 690 (5.40), 660 (4.62), 621 (4.65), 368 (4.95).

*1.1.4. Synthesis of compounds **1** and **2***

- *Synthesis of compound **1p**.*

4

+

Glycodendron 3

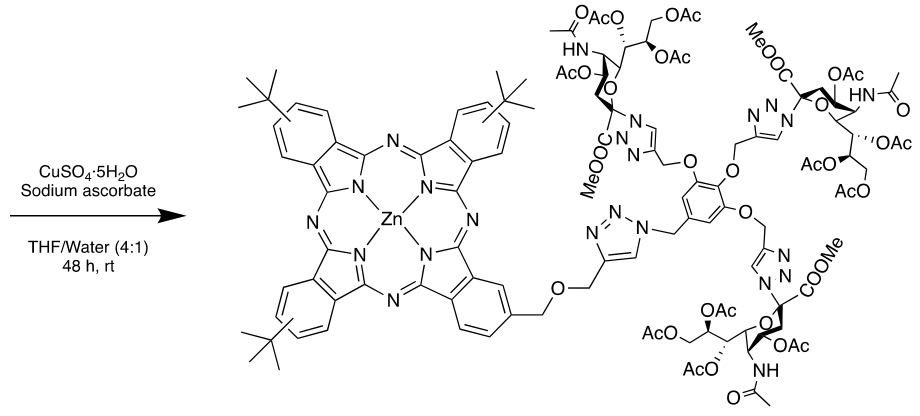

1p

Propargyloxy-ZnPc (**4**) (0.02 g, 0.024 mmol), glycodendron **3** (0.05 g, 0.03 mmol) and sodium ascorbate (1.43 mg, 7.26 μmol) were placed into a two-necked round bottom flask, under argon atmosphere. THF (4 mL) and distilled water (1 mL) were added with a syringe, and $CuSO_4 \cdot 5H_2O$ (0.18 mg, 7.26 μmol) was added. The reaction mixture was stirred for 48 h at rt. After that, the crude was treated with Quadrasil MP, to remove copper, filtered and evaporated. The product was purified through two consecutive SEC columns, with BioBeads as stationary phase and DCM and DMF as eluents, respectively. Yield: 31 % (0.02 g).

^{1}**H-NMR** (500 MHz, DMSO$_{d6}$): *δ* (ppm) = 9.3 (m, 8H; $H_{ar\ Pc}$), 8.4 – 8.3 (m, 6H; triazole, $H_{ar\ Pc}$), 8.2 (m, 2H; triazole, $H_{ar\ Pc}$), 7.8 – 7.7 (m, 3H; NH), 7.0 (s, 2H; H_{ar}), 5.57 (s, 2H; triazole-C<u>H</u>$_2$-H$_{ar}$), 5.2 – 4.9 (m, 19H; H4$_{SA}$, H8$_{SA}$, H7$_{SA}$, Pc-C<u>H</u>$_2$-O-C<u>H</u>$_2$, O-C<u>H</u>$_2$-triazole), 4.24 (d, J = 10.85, 3H; H9$_{SA}$), 4.1 – 3.9 (m, 9H; H9$_{SA}$, H6$_{SA}$, H5$_{SA}$), 3.8 – 3.7 (m, 9H; COOMe), 3.16 (dd, *J* = 12.45, *J* = 3.9 Hz, 3H; H3$_{SA,eq}$), 2.0 – 1.9 (m, 36H; CH_3-acetyl), 1.76 (s, 27H; CH3), 1.67 (s, 9H; CH_3CON).

HR-MS (ESI Positive, MeOH + 0.1% formic acid): Calc for $C_{124}H_{141}N_{23}Na_2O_{40}Zn$ $[M+2Na]^{2+}$: *m/z*: 1351.9405, found 1351.9423.

UV-Vis (DMF): λ_{max} (nm) (log *ε*) = 674 (5.09), 642 (4.26), 612 (4.29), 350 (4.65).

- *Synthesis of compound **1**.*

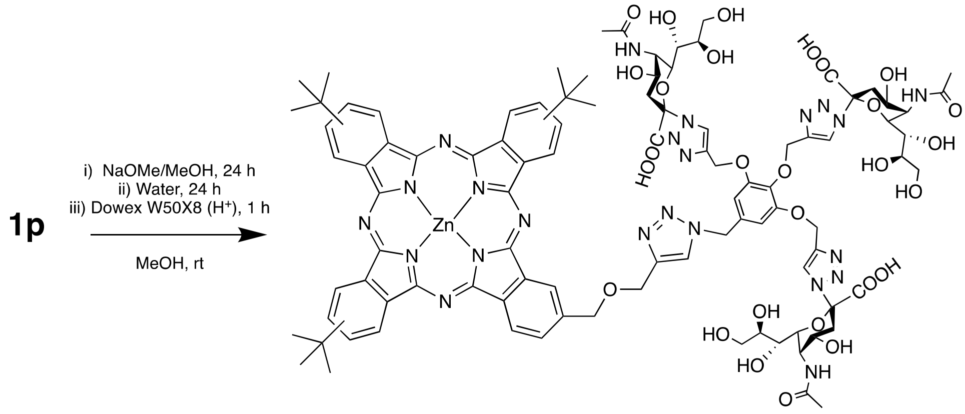

1p (15 mg, 5.64 μmol) was dissolved in dry methanol, and sodium methoxide in methanol (3 mL) was added dropwise at 0 ºC. The reaction mixture was stirred for 24 h at rt, and after that distilled water (3 mL) was added. The reaction mixture was stirred for an additional period of 24 h at rt, and after that the crude reaction was treated with Dowex, W50X8 (H^+), filtered, and the solvent was evaporated. To eliminate the generated acetic acid, the ZnPc material was crushed with acetone and filtered, to afford the pure compound as a dark blue fine powder in quantitative yield. Yield: 95% (11.8 mg).

HR-MS (ESI Negative, MeOH + 0.03% formic acid): Calc for $C_{97}H_{109}N_{23}O_{28}Zn$ $[M-2H]^{2-}$: *m/z*: 1054.8566, found 1054.8550.

UV-Vis (DMF): λ_{max} (nm) (log *ε)* = 675 (4.63), 641 (3.77), 615 (3.8), 345 (4.24).

- *Synthesis of compound* ***2p****.*

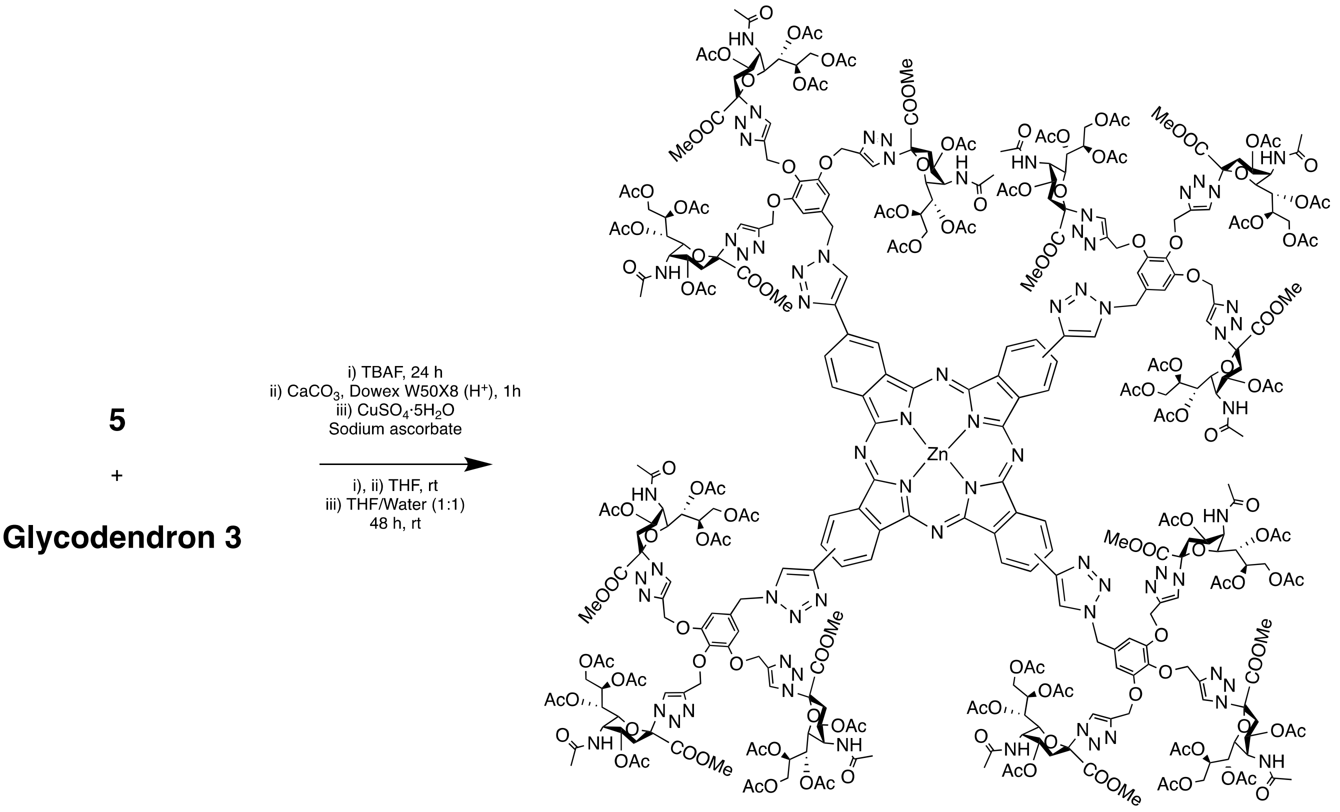

Symmetrically substituted tetraethynyl-ZnPc (**5**) (0.04 g, 0.035 mmol) was dissolved in 4 mL of dry THF, under argon atmosphere. TBAF (3 mL, 0.35 mmol) was added dropwise, and the reaction mixture was stirred for 24 h at rt. After that, the crude was treated for 1 h with $CaCO_3$ and Dowex, W50X8 (H^+) and filtered. The solvent was evaporated, and the compound was used in the next step without any further purification/characterization step, due to its poor solubility and low stability.

Deprotected tetraalkynyl-ZnPc (0.013 g, 0.02 mmol), glycodendron **3** (0.17 g, 0.09 mmol) and sodium ascorbate (4 mg, 0.023 mmol) were placed into a two-necked round bottom flask, under argon atmosphere. THF (2 mL) and distilled water (2 mL) were added with a syringe, and $CuSO_4·5H_2O$ (5 mg, 0.023 mmol) was added. The reaction was stirred for 48 h at rt. After that, the crude was treated with Quadrasil MP, to remove copper ions, filtered and evaporated. The product was purified by SEC chromatography, using BioBeads as stationary phase and DMF as eluent. Yield: 26 % (0.04 g).

^{1}H-NMR (500 MHz, DMSO-d_6): δ (ppm) = 9.98 (s, 4H; $H_{ar\,Pc}$), 9.6 – 9.5 (m, 4H; $H_{ar\,Pc}$), 9.3 – 9.2 (m, 4H; $H_{ar\,Pc}$), 8.79 (s, 4H; $triazole_{Pc}$), 8.41 (s, 8H; $triazole_{SA}$), 8.26 (s, 4H; $triazole_{SA}$), 7.75 (d, J = 7.05 Hz, 12H; NH), 7.16 (s, 8H; H_{ar}), 5.77 (s, 8H; triazole-C<u>H</u>$_2$-H_{ar}), 5.3 – 5.0 (m, 60H; $H4_{SA}$, $H8_{SA}$, $H7_{SA}$, O-C<u>H</u>$_2$-$triazole_{SA}$), 4.3 – 4.2 (m, 12H; $H9_{SA}$), 4.1 – 4.0 (m, 36H; $H9_{SA}$, $H5_{SA}$, $H6_{SA}$), 3.8 – 3.7 (m, 36H; COOMe), 3.2 (m, 12H; $H3_{SA,eq}$), 2.60 (m, 12H; $H3_{SA,ax}$), 2.0 – 1.9 (m, 144H; CH_3-acetyl), 1.67 (s, 36H; CH_3CON).

HR-MS (ESI Positive, MeOH + 0.1% formic acid): Calculated for $C_{344}H_{404}N_{68}Na_4O_{156}Zn$ $[M+4Na]^{4+}$: *m/z*: 2035.8692, found 2035.8692.

UV-Vis (DMF): λ_{max} (nm) (log ε) = 689 (5.11), 661 (4.34), 621 (4.34), 361 (4.68).

- *Synthesis of compound **2**.*

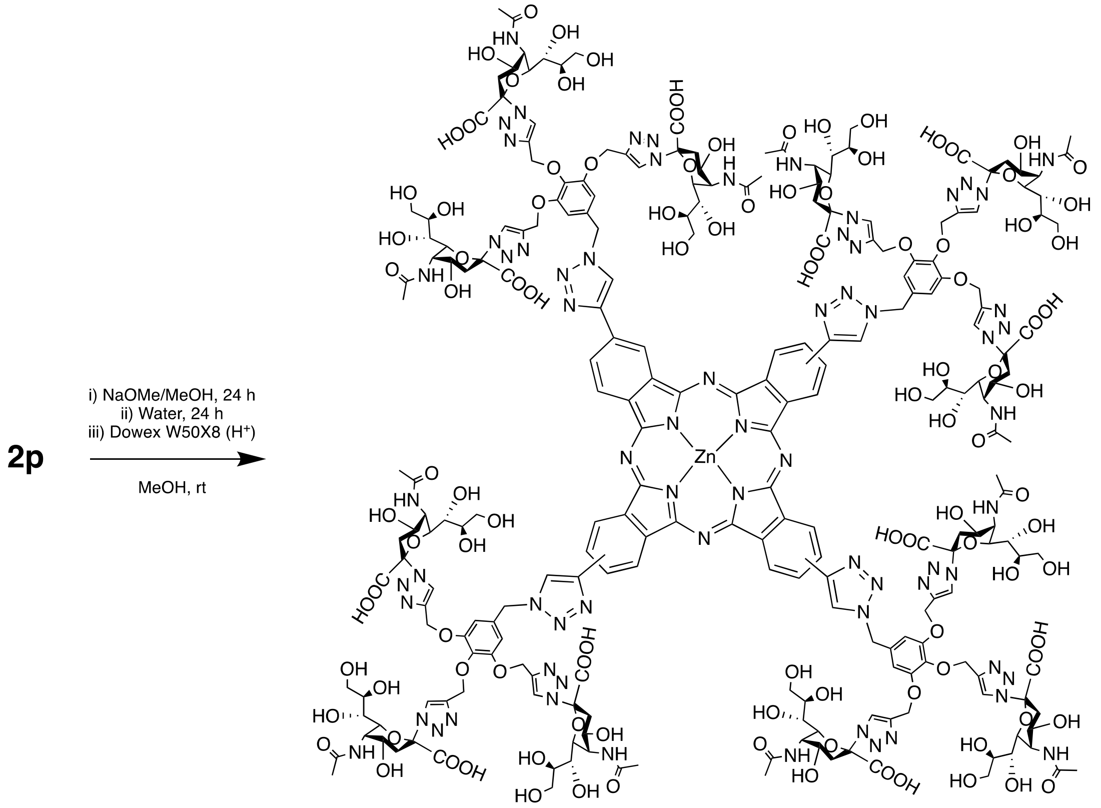

2p (0.035 g, 4.34 mmol) was dissolved in dry methanol, and sodium methoxide in methanol (3 mL) was added dropwise at 0 ºC. The reaction mixture was stirred for 24 h at rt, and after that distilled water (3 mL) was added. The reaction was stirred for an additional period of 24 h at rt, and the reaction crude was treated with Dowex, W50X8 (H^+), filtered, and the solvent was evaporated. To eliminate the generated acetic acid, the ZnPc material was crushed with acetone and filtered off, to afford the pure compound as a dark green fine powder in quantitative yield. Yield: 95% (0.025 g).

HR-MS (ESI Negative, MeOH + 0.1% formic acid): Calc for $C_{236}H_{277}N_{68}Na_3O_{108}Zn$ $[M-7H+2Na]^{5-}$: *m/z*: 1180.9491, found 1180.9449.

UV-Vis (DMF): λ_{max} (nm) (log *ε)* = 689 (4.93), 670 (4.33), 626 (4.15), 365 (4.51).

1.2. Characterization of the ZnPc-SA biohybrid precursors (the spectroscopic characterization of compounds **1/1p** and **2/2p** is given in section 2).

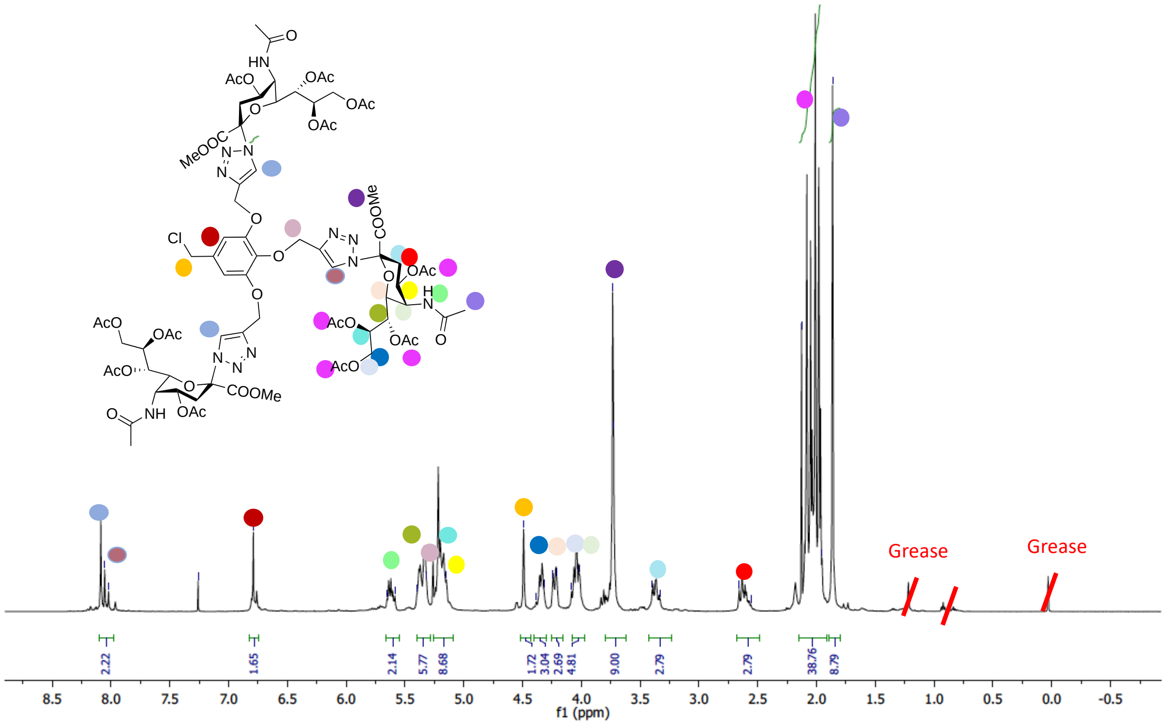

^{1}H-NMR of **S3** in $CDCl_3$

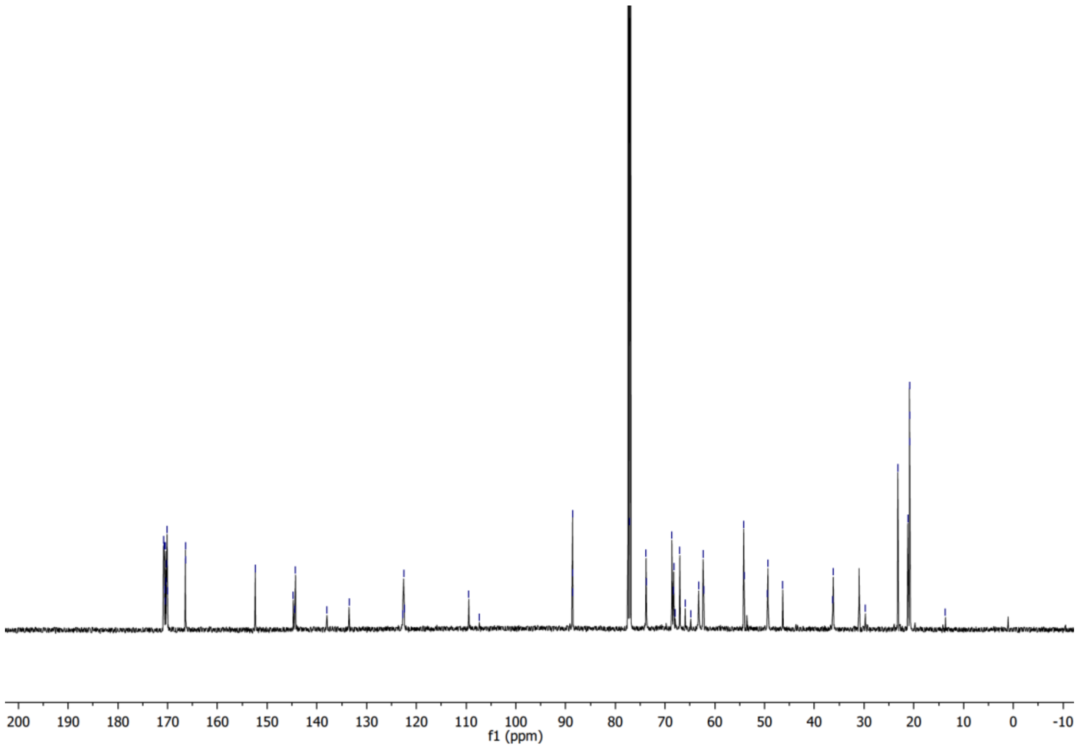

^{13}C-NMR of **S3** in $CDCl_3$

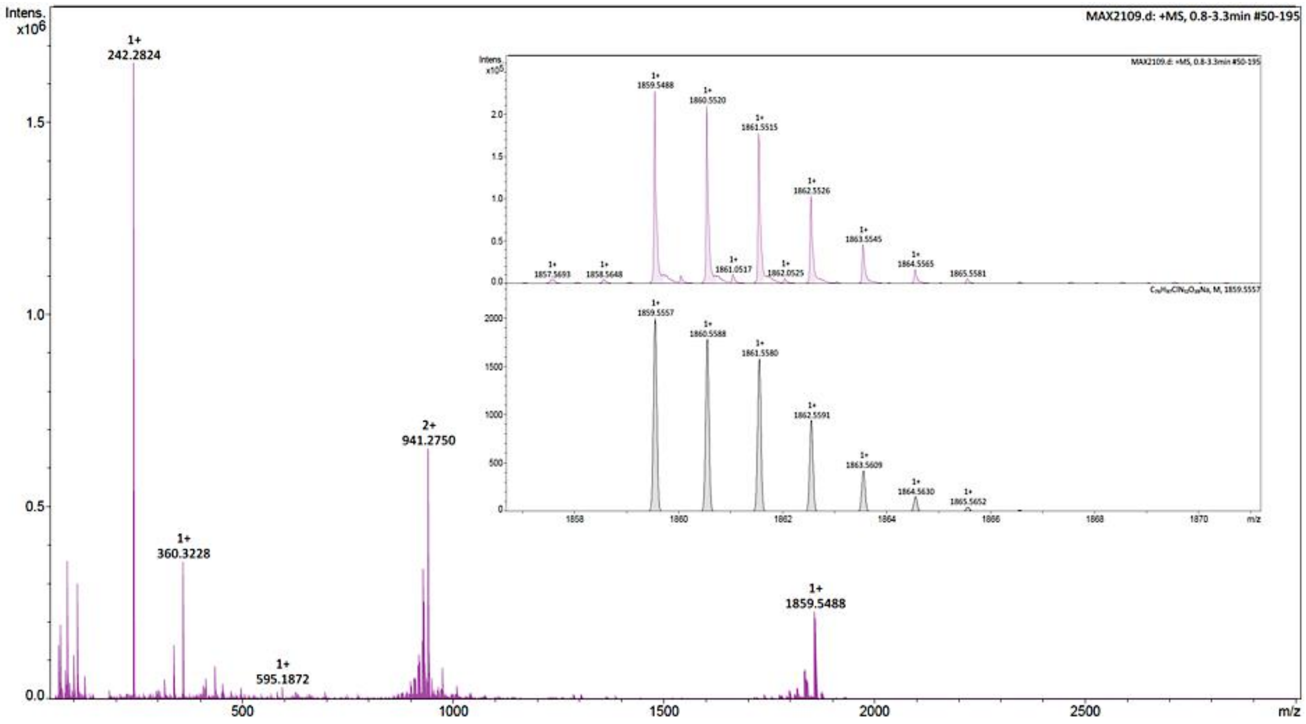

Mass spectra (ESI) of **S3**

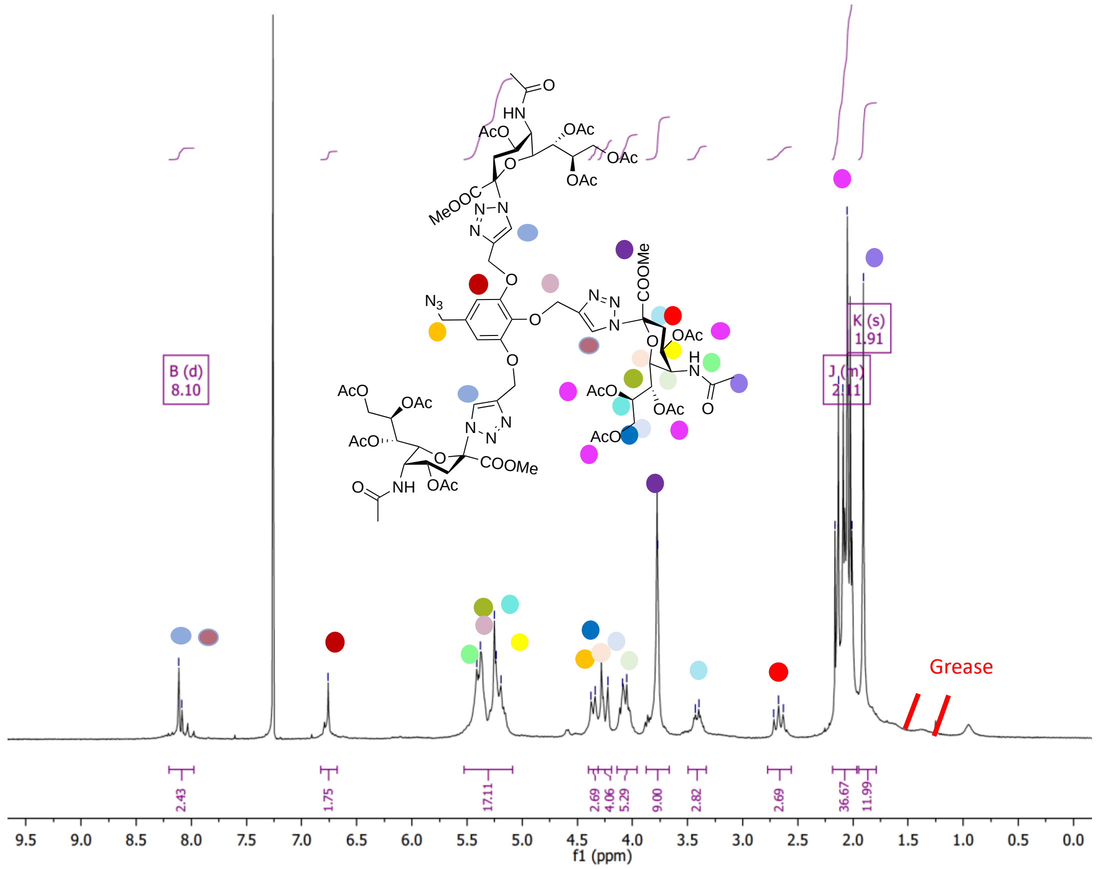

^{1}H-NMR of glycodendron **3** in $CDCl_3$

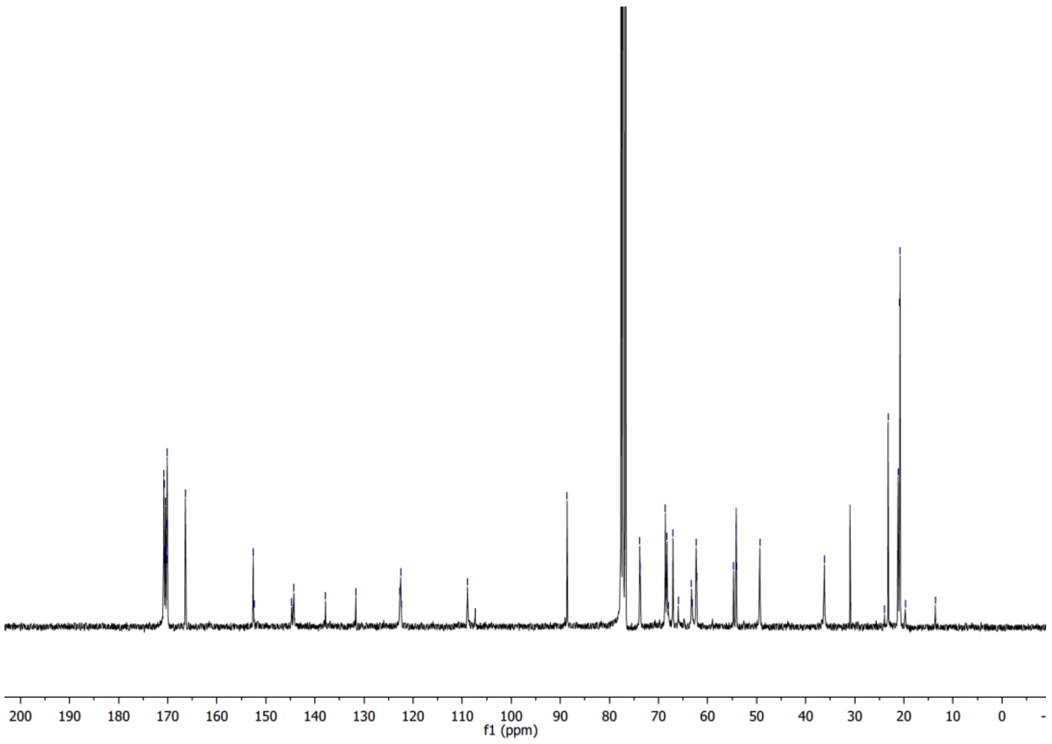

^{13}C-NMR of glycodendron **3** in $CDCl_3$

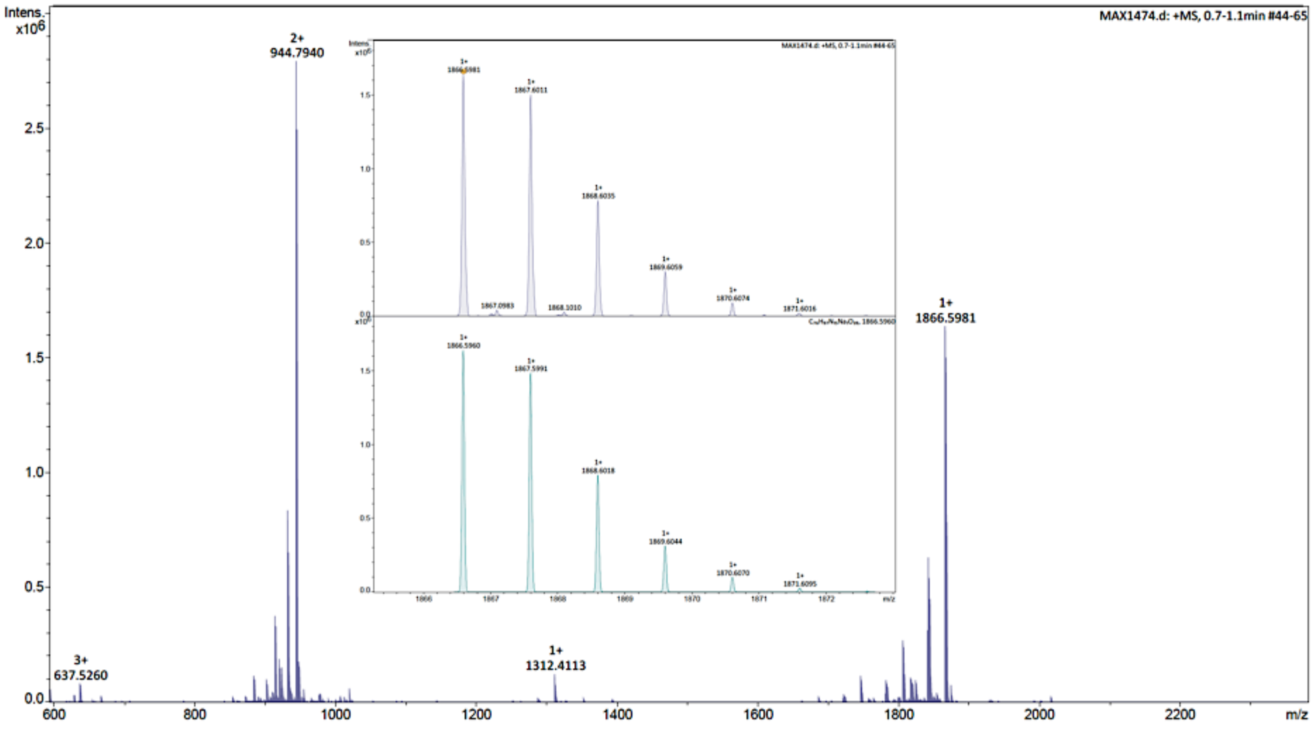

Mass spectra (ESI) of glycodendron **3**

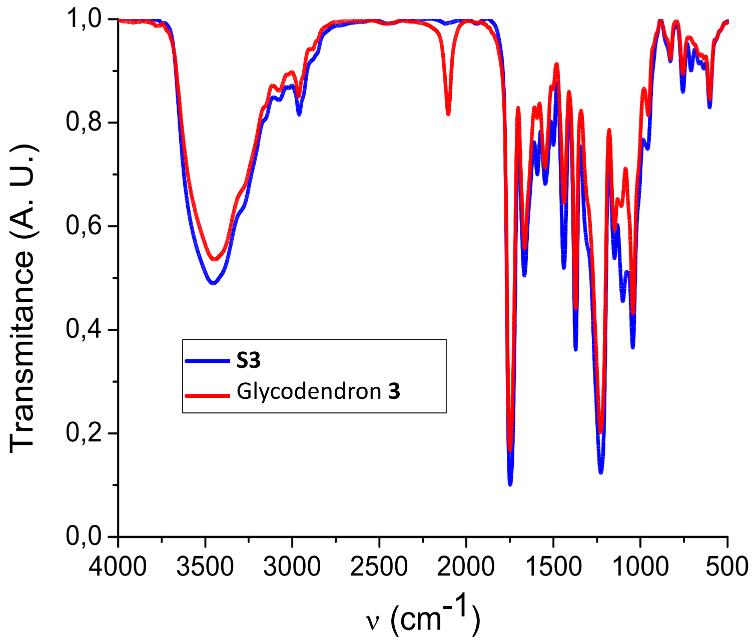

IR spectra of **S3** and glycodendron **3**

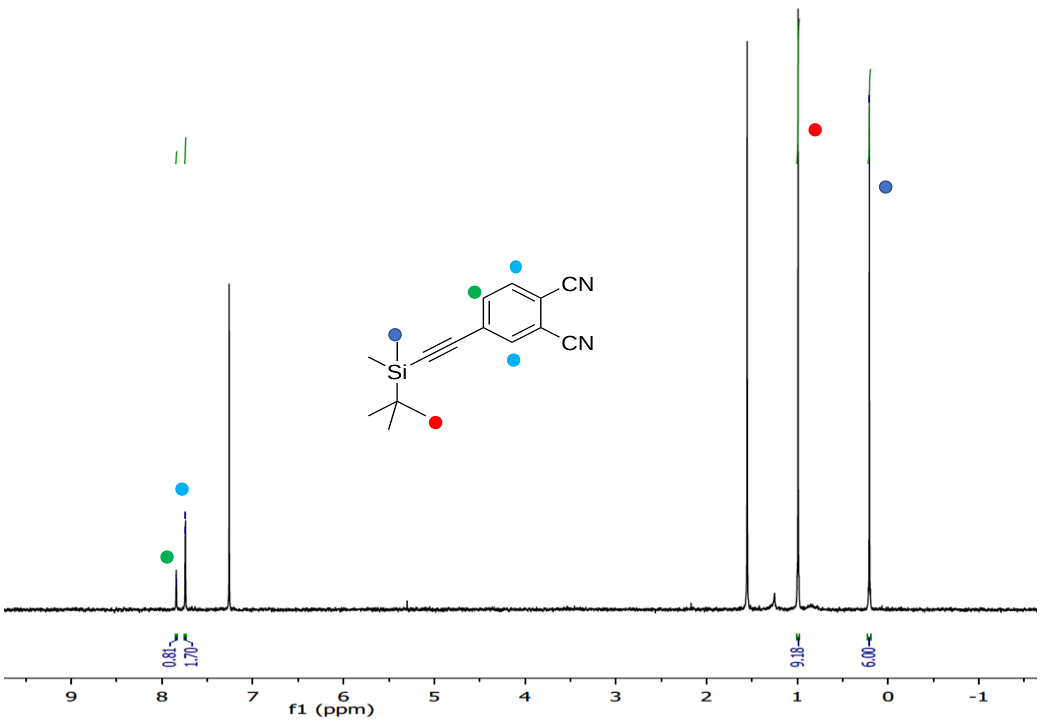

^{1}H-NMR of **S5** in $CDCl_3$

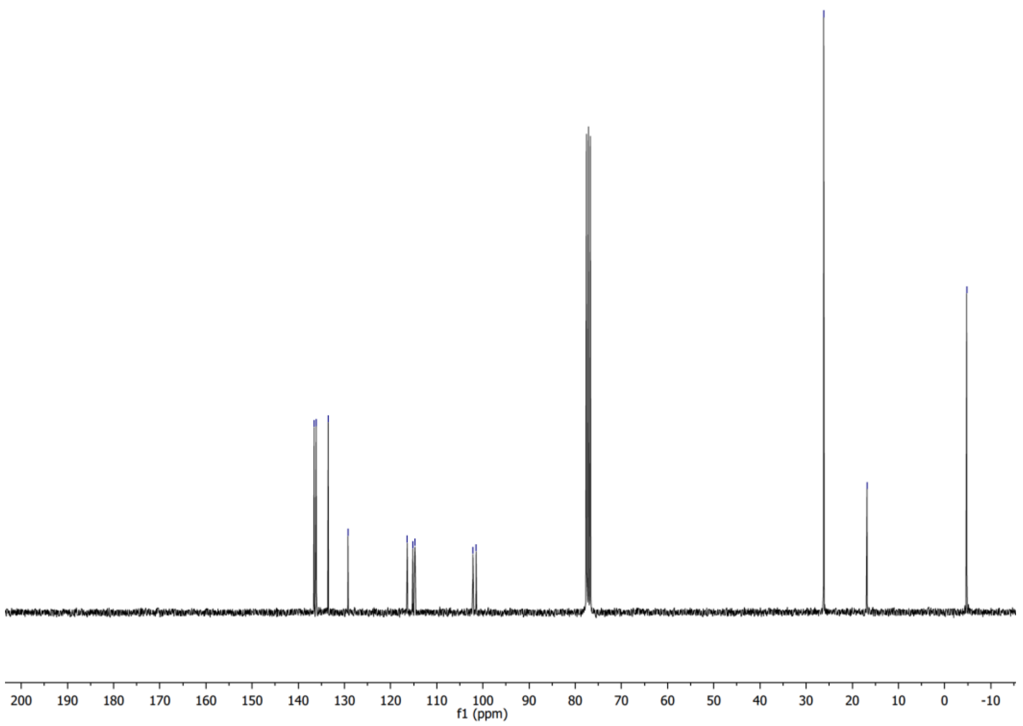

^{13}C-NMR of **S5** in $CDCl_3$

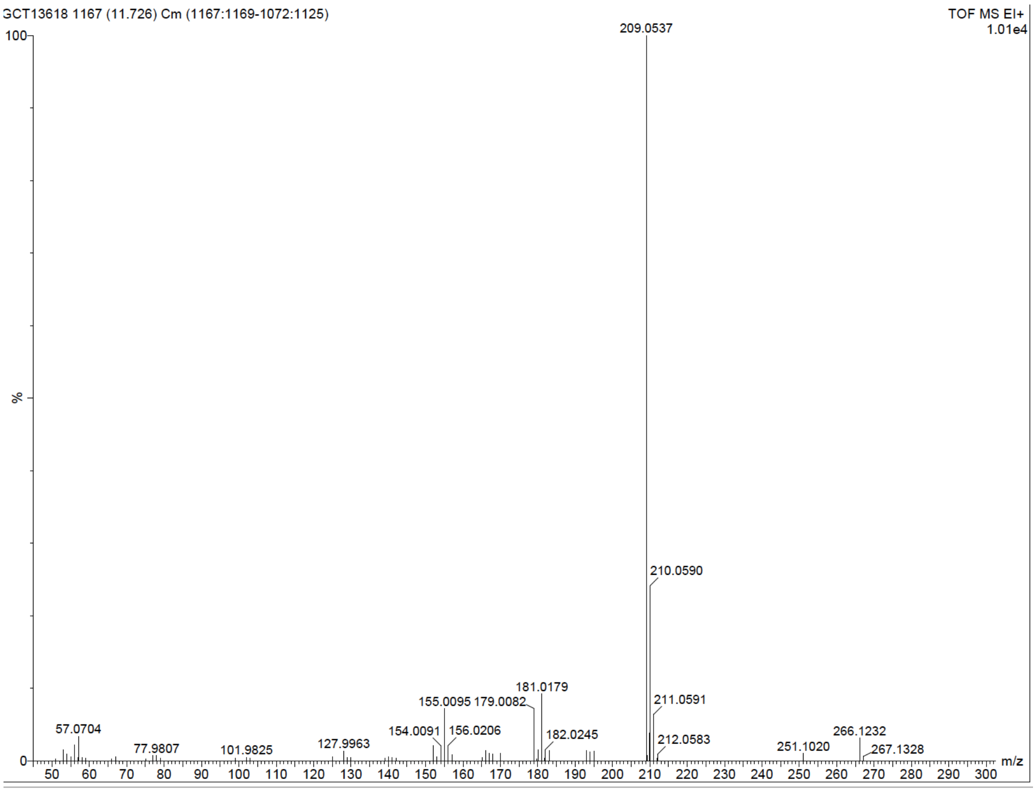

Mass spectra (EI positive) of **S5**

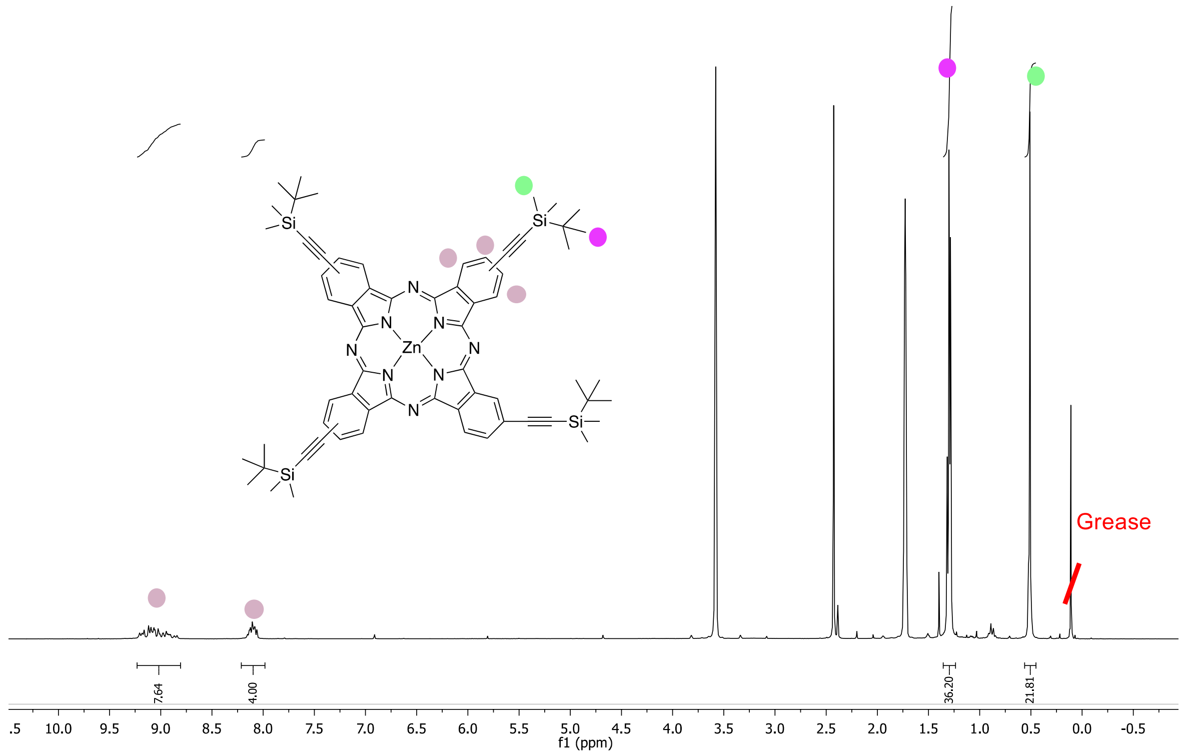

^{1}H-NMR of **5** in THF-d8

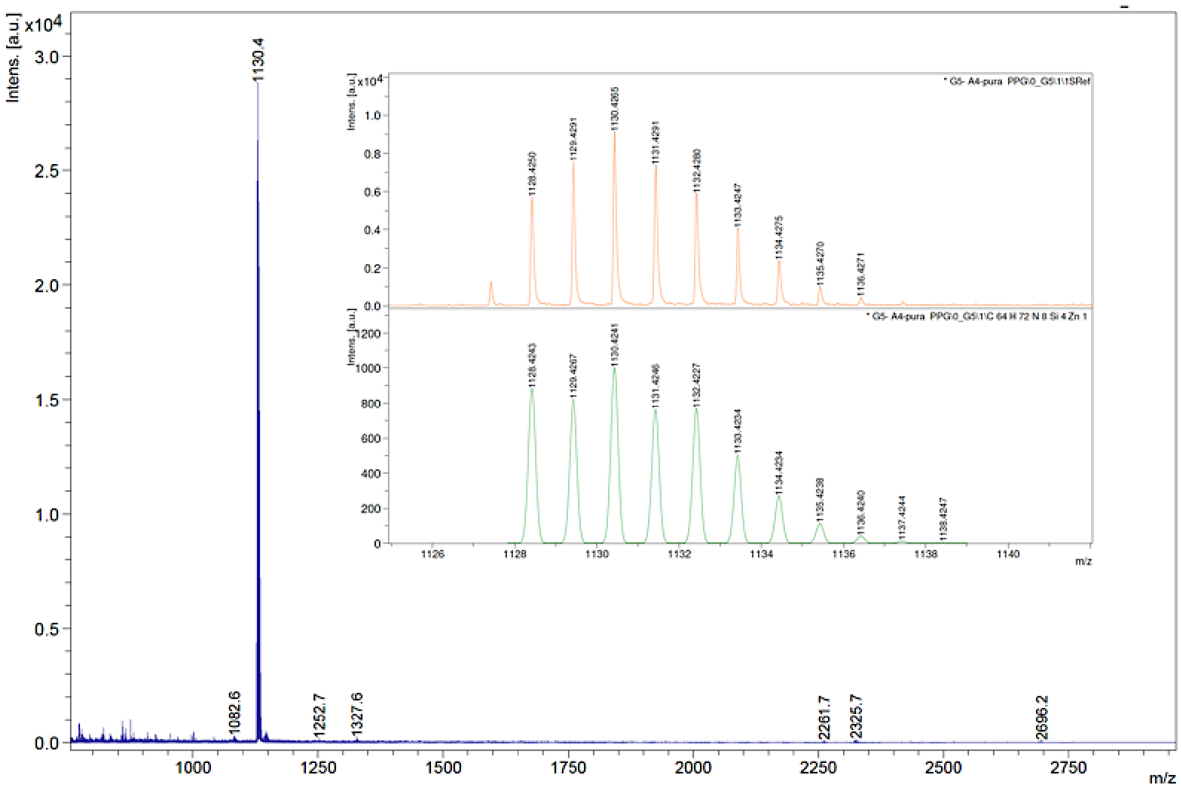

Mass spectra (MALDI-TOF) of **5**

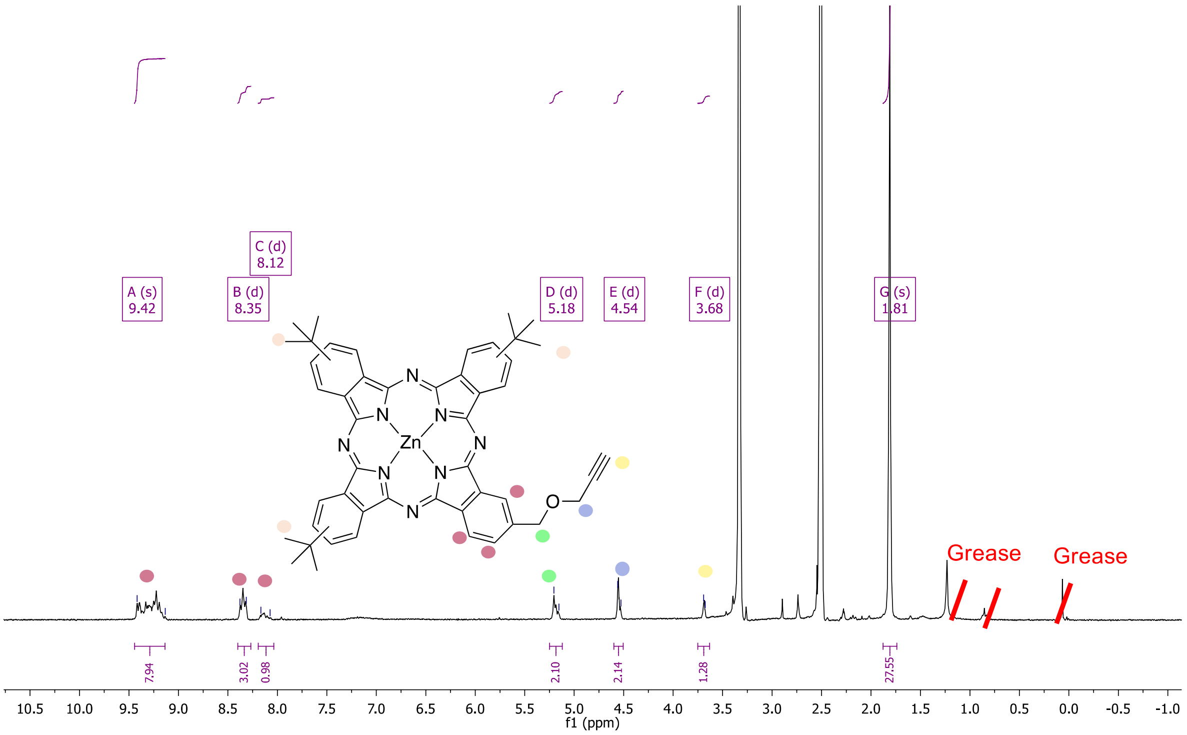

^{1}H-NMR of **4** in DMSO-d6

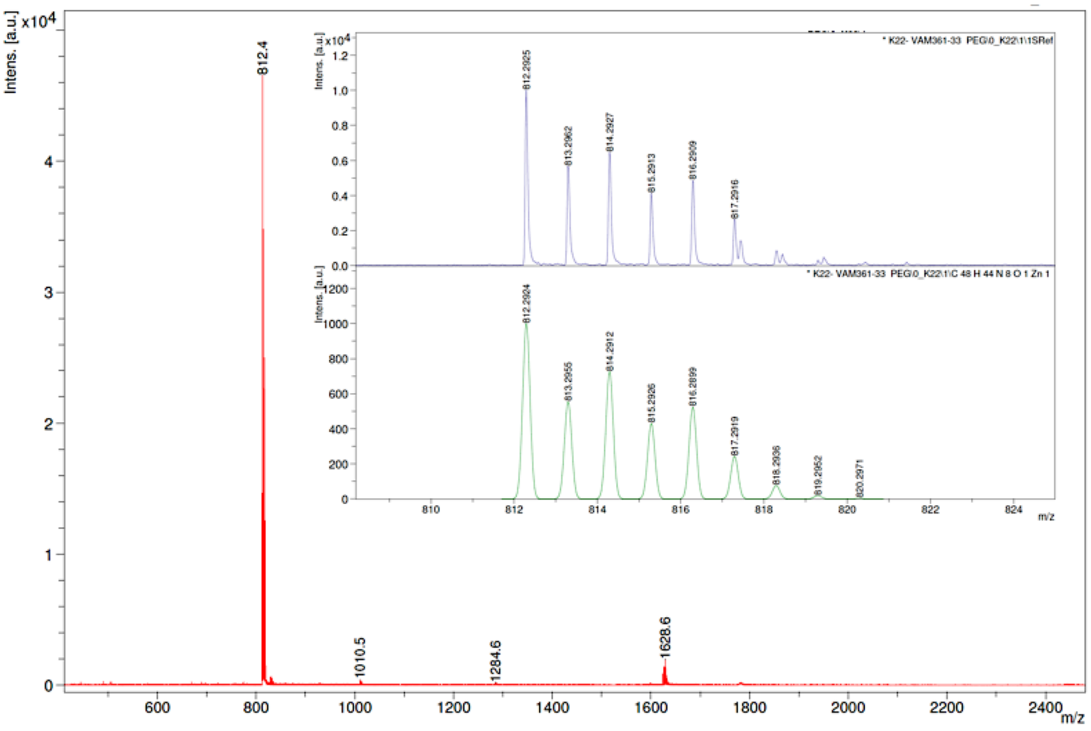

Mass spectra (MALDI-TOF) of **4**

1.3. Fluorescence and singlet oxygen measurements.

Fluorescence quantum yields. Φ_F values were measured by the method of Williams,[6] using the following equation:

$$\phi_F^S = \phi_F^R \left(\frac{Quot_S}{Quot_R}\right)\left(\frac{\eta_S^2}{\eta_R^2}\right)$$

Where Φ_F is the fluorescence quantum yield, *S* is the sample, *R* is the reference. *Quot* is the quotient of the integrated fluorescence intensity and the absorption at the excitation wavelength of the sample. h is the refractive index of the solvent. Φ_F of ZnPc in DMF is 0.28.[7] **1** and ZnPc were irradiated at 588 nm, while **2** and ZnPc were irradiated at 612 nm.

Singlet oxygen quantum yields. Φ_Δ values have been calculated using the *relative method*, where a scavenger, in this case 1,3-diphenylisobenzofuran (DPBF), is decomposed by the presence of $^1O_2$.[8] The compound that was used as reference was non-substituted ZnPc, which has a $\Phi_{\Delta(DMF)} = 0.56$.[9] To carry out the procedure, 3 mL of a stock solution of DPBF (A = 1) in DMF were placed into a 10 x 10 mm quartz optical cell, which was bubbled for 1 min with $^3O_2$. A solution of **1** or **2** was added, having an absorbance in the maximum of Q band around 0.1. Using a halogen lamp (300W), the mixture was irradiated under stirring, for defined intervals of time, with the duration that was necessary for detecting a decrease of the absorbance of DPBF of 3 - 4% at 414 nm. Light was filtered using neutral density filters (FBS-ND03 or FB-ND10) when it was necessary, and a filter to remove light under 530 (Newport filter FSQ-OG530) and a water filter of 6 cm. Experiments were done per triplicate, and final results are expressed as the average of them.

The Φ_Δ values of **1** and **2** were calculated using the following equation:

$$\phi_\Delta^S = \phi_\Delta^R \frac{k^S I_{aT}^R}{k^R I_{aT}^S}$$

Where Φ_Δ is the singlet oxygen quantum yield, *S* is the sample, *R* is the reference, *k* is the slope of a plot of $ln(A_0/A_t)$ versus irradiation time, and A_0 and A_t the absorbance of DPBF before and after irradiation time (t) respectively. I_{aT} is the total amount of light that the dye absorbs and is calculated as a sum of intensities of the absorbed light I_a at wavelengths from the filter cutoff to 800 nm (step 0.5 nm). I_a at one determined wavelength can be determined by Lambert-Beer law:

$$I_a = I_0(1 - e^{-2.3A})$$

Where *A* is the absorbance of the photosensitizer at the determined wavelength, and I_0 the transmittance of the filter at the same wavelength.

1.4. Determination of partition coefficients in octanol/water (log $P_{o/w}$).

Equal volumes of *n*-octanol and water were stirred for 3 days at rt, promoting the other solvent saturation in both phases. PS **1** and PS **2** were added from a water stock solution to 2 mL of the solvents mixture (%water < 1%, [PS] = 10^{-5} M) and the mixture was stirred for 30 min and later incubated for 1 h at rt. Once both phases were separated, and 10 μL from each phase were taken and diluted in DMF to 1.01 mL. The UV-Vis spectra of the resulting solutions in DMF were recorded (see Figure S10), and the partition coefficient was calculated using the absorbance values at the Q-band maximum absorption. The results are expressed as the average of three independent measurements, following the equation below:[10]

$$log\, P_{OW} = log\left(\frac{A(DMF)_O \cdot V_W}{A(DMF)_W \cdot V_O}\right)$$

1.5. Supramolecular polymerization studies

The degree of aggregation (α_{agg}) has been calculated using the following equation:[11]

$$\alpha_{agg} = \frac{A_{exp}-A_{min}}{A_{max}-A_{min}} \quad (1)$$

where A_{exp} is a chosen experimental value of absorbance, while A_{max} and A_{min} are the maximum and minimum absorbance values, respectively. If it is considered a two-state equilibrium, there is a sigmoidal relation between the degree of polymerization or the molar fraction of aggregated species α_{agg}(T) and temperature. α_{agg}(T) allows determining the number-averaged degree of polymerization DP_N(T) by the following equation:

$$DP_N = \frac{1}{\sqrt{1-\alpha_{agg}(T)}} \quad (2)$$

Where:

$$\alpha_{agg} = 1 - \frac{2Kc_T+1-\sqrt{4K(T)c_T+1}}{2K^2c_T^2} \quad (3)$$

The total concentration of molecules c_T and the equilibrium constant *K* are related to DP_N through the equation:

$$DP_N = \frac{1}{\sqrt{1-\alpha_{agg}(T)}} = \frac{1}{2} + \frac{1}{2}\sqrt{4K(T)c_T + 1} \quad (4)$$

(4) is equal to:

$$DP_N = \frac{c_T}{c_N} = \frac{c_T(1-Kc_1)}{c_1} = \frac{1+\sqrt{4Kc_T+1}}{2} \quad (5)$$

1.6. Subcellular localization and phototoxicity studies

Cell Cultures. For in vitro studies, three human tumoral cell lines were used: SCC-13 (squamous cell carcinoma from face), A431 (squamous cell carcinoma from vulva), and HeLa (cervical adenocarcinoma). Cell lines were grown in DMEM (Dulbecco's modified Eagle's medium high glucose) supplemented with 10% (v/v) fetal bovine serum (FBS), 100 units/mL penicillin, and 100 μg/mL streptomycin, all from Thermo Fisher Scientific Inc. Cell

cultures were performed under standard conditions of 5% CO2, 95% of humidity, and 37 °C of temperature and propagated by trypsinizing cultures with 1×10^{-3} M EDTA/0.25% Trypsin (w/v).

Photosensitizer Incubation. Stock solutions of **1** and **2** (1.05×10^{-3} M) were prepared in distilled water and working solutions were obtained in DMEM without FBS. All the treatments were performed when cultures reached around 60–70% of confluence.

Subcellular Localization. To analyze the intracellular localization of **1** and **2**, cells lines were grown on coverslips and, incubated with **1** and **2** to a final concentration of 10 µM for 18 h at 37 °C. After incubation, cells were further incubated for another 15 min with known fluorescent probes for lysosomes (LysoTracker Green DND-26, Invitrogen) at the concentration indicated by the suppliers. Then, cells were briefly washed in PBS, mounted on slides with a drop of PBS and immediately observed under the fluorescence microscope. The mitochondria were observed by its autofluorescence.

Photodynamic Treatment. Cells grown in 24-well plates were incubated with different concentrations of **1** and **2**, ranging from 0.1 to 10 micromolar (in DMEM without FBS) for 5 h. Subsequently, cells were irradiated with a red light emitting diode source (λ = 635 nm) for variable doses, ranging from 1 to 49 J/cm^2. After irradiation, cells were further incubated in complete medium at 37 °C for 24 h until evaluation. Dark control experiments were carried out in parallel, incubating the cells with the same concentrations of **1** and **2**, for 5 h in dark. In the same way, to test the effect of red light alone, cells were subjected to different light doses.

Morphological Changes and Cellular Toxicity. Changes in general cell morphology after 24 h of photodynamic treatments were analyzed by phase contrast microscopy. The toxicity of **1** and **2** on cells grown in monolayer was evaluated 24 h after photodynamic treatment by the MTT (3-(4,5-dimethylthiazol-2-yl)- 2,5-diphenyltetrazolium bromide, Sigma) assay. MTT solution (1 mg/mL) in PBS was diluted in DMEM (10% FBS) to a final concentration of 100 µg/mL, added to each well and incubated at 37 °C for 3 h. After incubation, the formazan crystals were dissolved in DMSO and the absorbance at 542 nm was measured through spectrophotometry (Espectra Fluor 4, Tecan). Cellular toxicity was expressed as cell survival percentage of control (cell survival (%) = (mean OD value of PDT treated cells/mean OD value of control cells) × 100%).

Intracellular ROS generation. The intracellular production of ROS in SCC-13 cells was evaluated by fluorescence microscopy. Cells were incubated with **1** and **2** at 0.5 µM for 5 h, and in the last hour 2,7-dichloro-dihydrofluorescein diacetate (DHF-DA, Abcam) was added to the cultures, reaching a final concentration of 6 µM. Afterwards, and without removing DHF-DA, cells were exposed to red light (9 J/cm^2), and analyzed immediately after irradiation by fluorescence microscopy, under blue excitation light (λ_{exc} = 436 nm). The corresponding controls were performed: cells incubated with DHF-DA without PS nor exposed to red light, and cells incubated with PS and DHF-DA but not exposed to red light. ROS production was quantified by using Image J after measuring green fluorescence.

Microscopy and Statistical Analysis. Microscopic observations were carried out using an Olympus BX61 epifluorescence microscope, equipped with a HBO 100 W mercury lamp and the corresponding filter sets for fluorescence microscopy: blue (450–490 nm, exciting filter BP 490), and green (545 nm, exciting filter BP 545). Photographs were obtained with digital camera Olympus DP50 and processed using Adobe PhotoShop CS5 extended version 12.0 software (Adobe Systems Inc., USA). The statistical significance was determined using t test and analysis of variance (ANOVA) followed by Bonferroni's test, and $p < 0.05$ was considered statistically significant. Data are expressed as the mean value ± standard deviation (SD) of at least three independent experiments.

2. Figures and Tables

2.1. Characterization of compounds 1p/1 and 2p/2

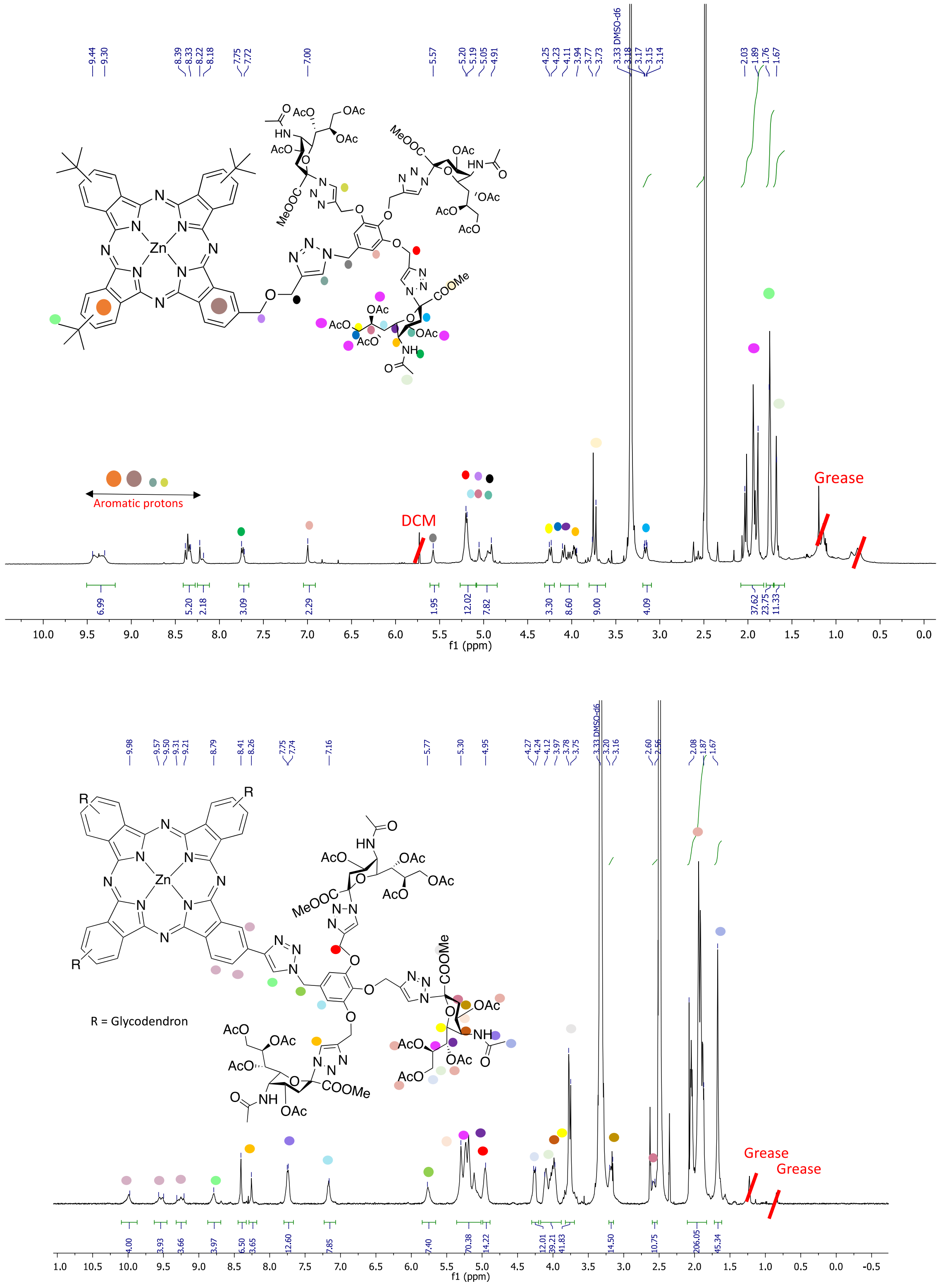

Figure S1. ^{1}H-NMR spectra of (top) **1p** and (bottom) **2p** in DMSO-d$_6$.

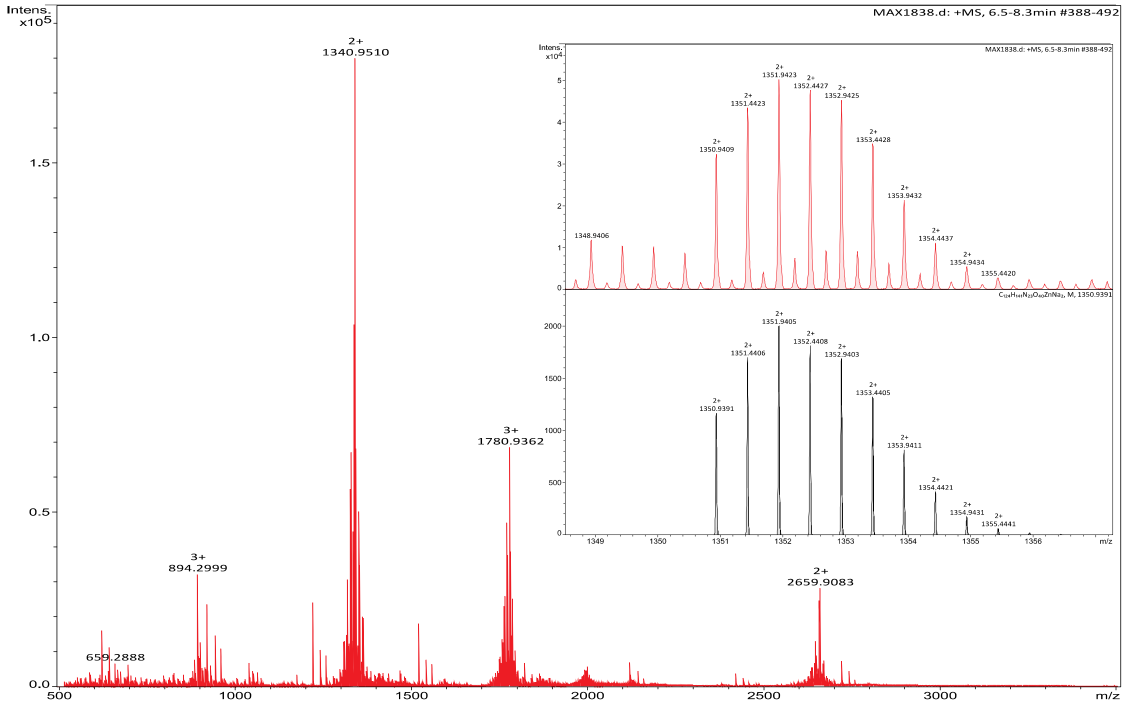

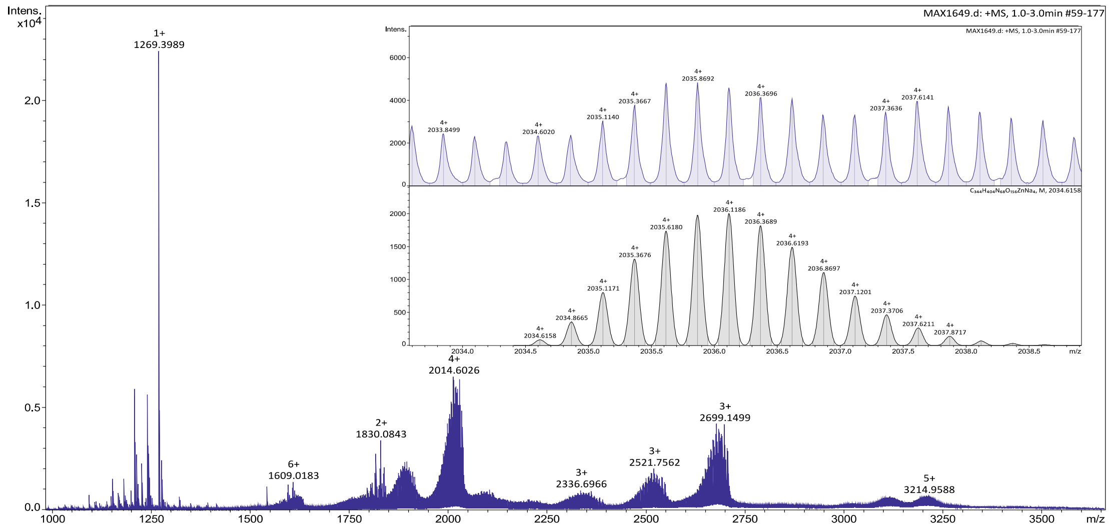

Figure S2. ESI mass spectra of (top) **1p** and (bottom) **2p.**

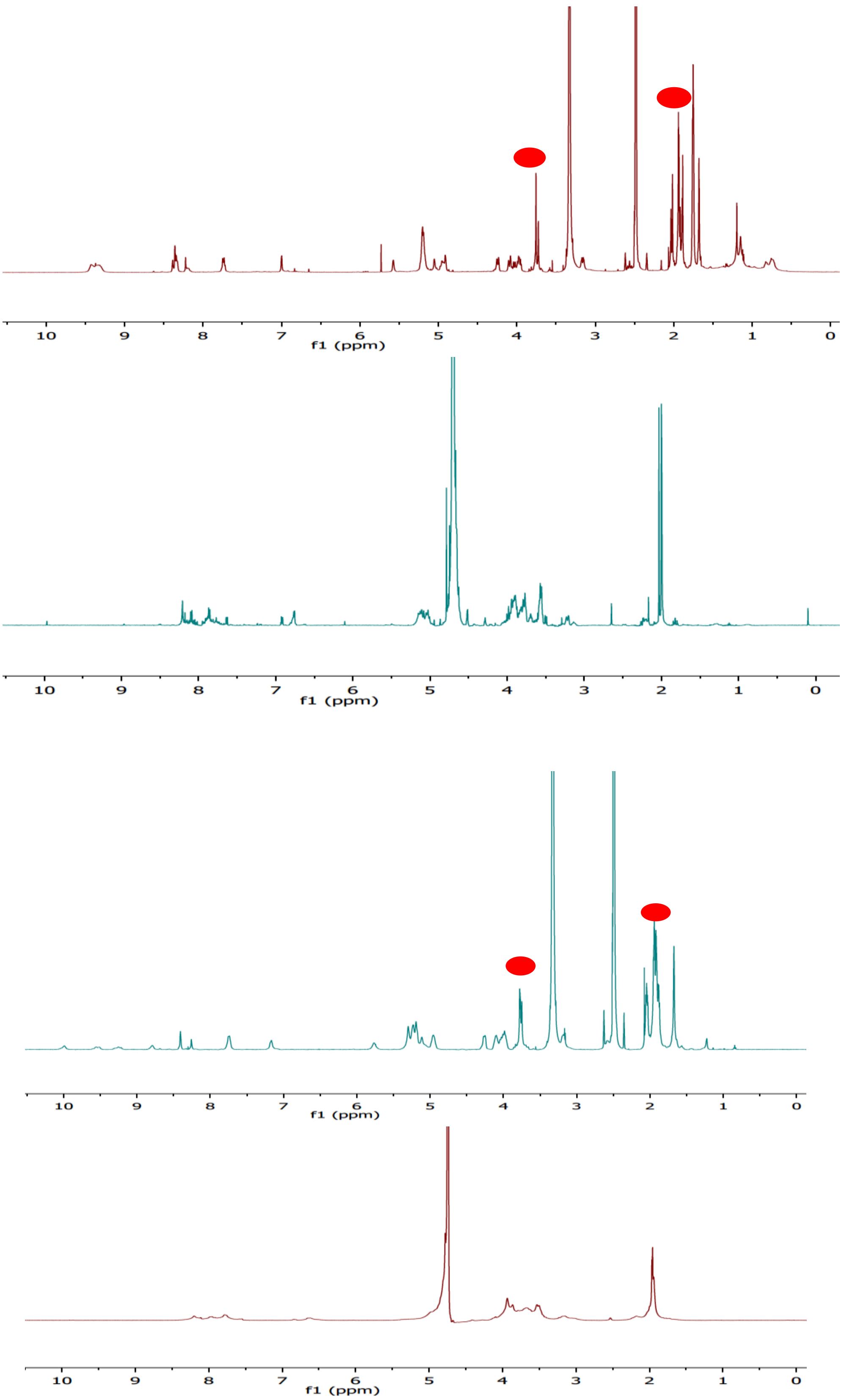

Figure S3. Comparison of the ^{1}H-NMR spectra of **1p** with **1** (top) and **2p** with **2** (bottom) in DMSO-d_6 and D_2O, respectively. Red points indicate the presence of protecting groups in the two precursors, which are absent in the final products.

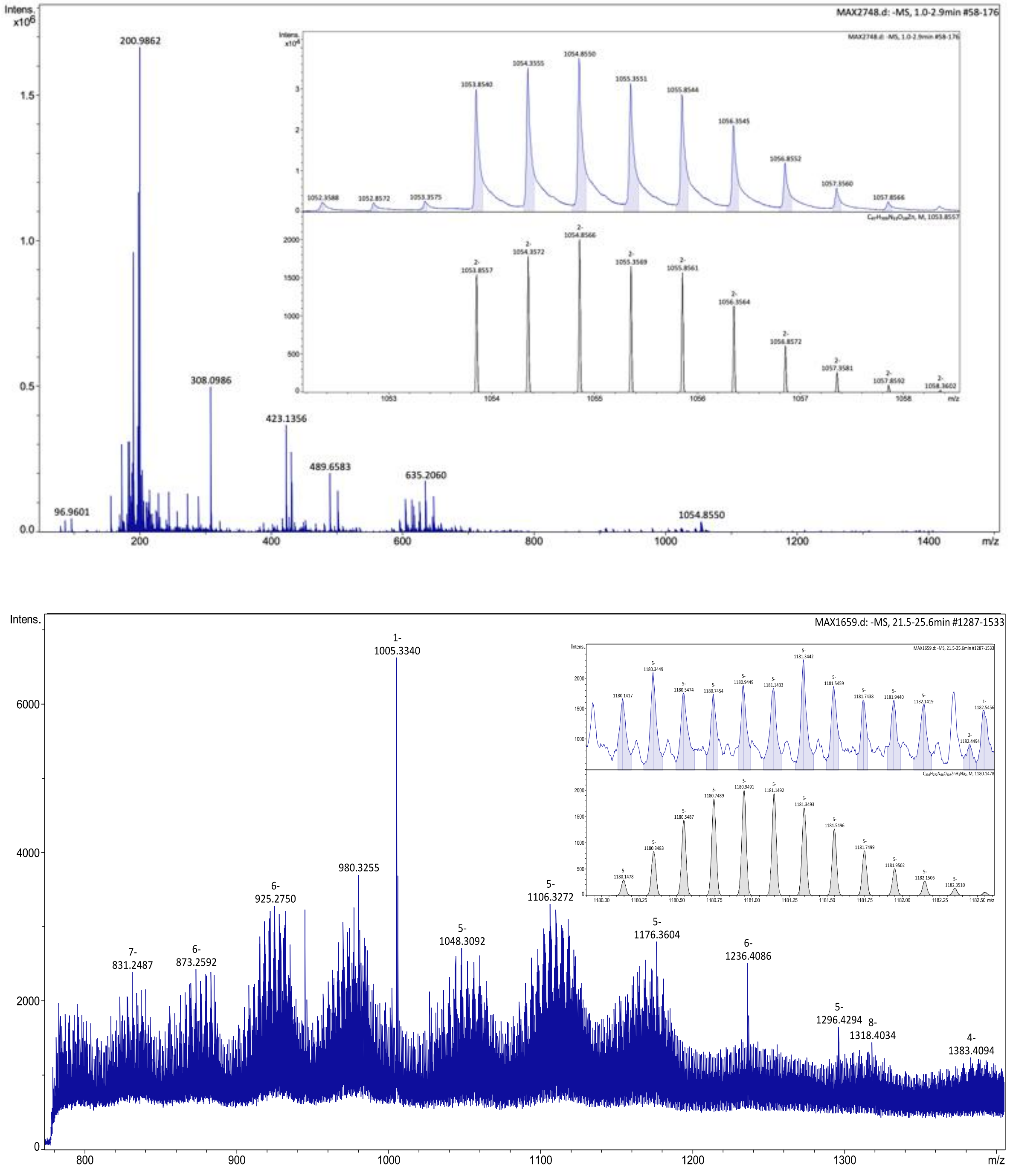

Figure S4. ESI mass spectrum of (top) **1** and (bottom) **2.**

2.2. HPLC characterization

HPLC was recorded in a HPLC Agilent 1100 Series system with a 200-C18-42 (ACE 3 C18-AR, 150x3mm, 3µm) column, using a water/acetonitrile gradient and a flow of 0.5 mL/min. Under these conditions, the PS **2** elutes as a single peak with a water/acetonitrile (60:40) mobile phase, the retention time being 0.92 min (Figure S5 bottom). PS **1**, in turn, eluted with pure acetonitrile as eluent (Figure S5 top), as a mixture of 8 regioisomers, which is expected theoretically for tri-terc-butyl asymmetrically substituted ZnPc with the 4 substituents at the β positions of each isoindole.[12] The HPLC separation of Pc regioisomers has actually been demonstrated previously.[13] The UV-Vis spectrum for all these peaks showed a perfectly superimposable Q-band (inset), demonstrating their identical chemical nature (differing only in the regiochemical position of the substituents). The band at higher wavelengths must arise from aggregation or interaction with the column, which could have helped enhancing the splitting/separation of peaks. The observed retention times were 6.43 (1 regioisomer), 7.22 (broader, 2 regioisomers), 8.17, 8.58, 8.97, 9.31 and 9.61 (5 regioisomers). The relative areas of the three main groups of regioisomers were 20.9, 47.1 and 32%. Importantly, it is clear from the comparison with the retention time of **2** (and the polarity of the eluent required to elute it) that hybrid **1** is much less hydrophilic, which could explain their different tendency to get inserted into membranes.

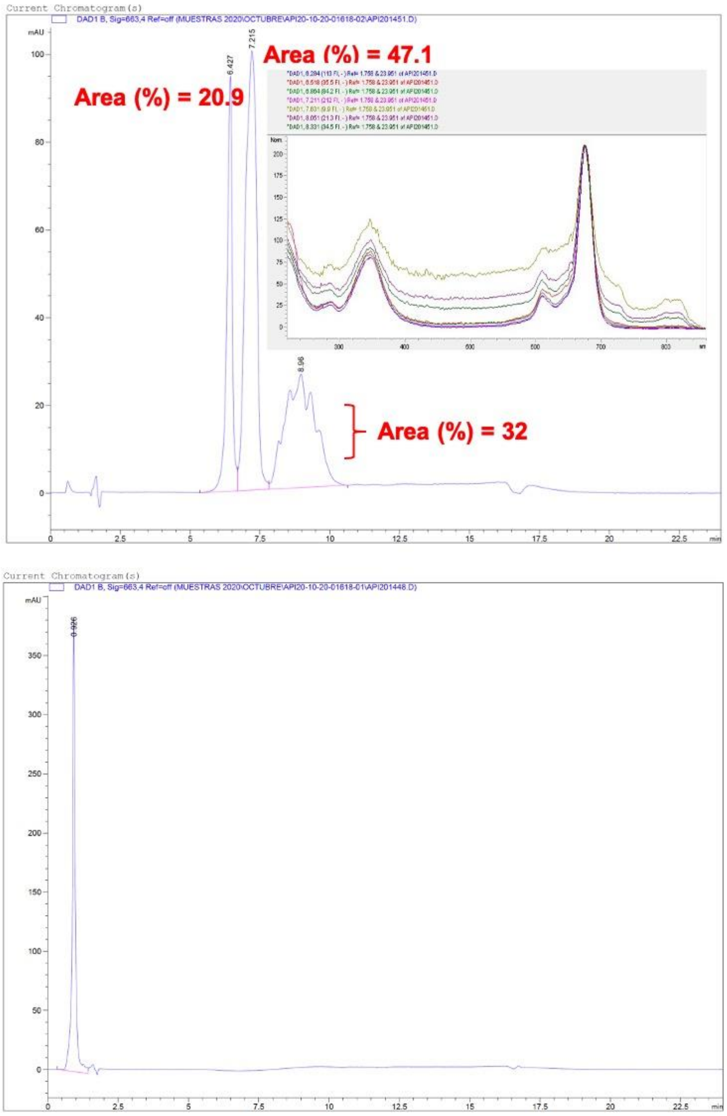

**Gradient:** Phase A, water Phase B, acetonitrile		
**Time / min**	**% Phase A**	**% Phase B**
0	60	40
2.5	60	40
7	0	100
13	0	100
14	60	40
24	60	40

Figure S5. HPLC chromatograms of **1** (top) and **2** (bottom). Eluting solvents = water/acetonitrile; flow rate = 0.5 mL/min; temperature = 25 ºC; detection wavelength = 663 nm; injection volume = 10 µL.

2.3. Photochemical characterization

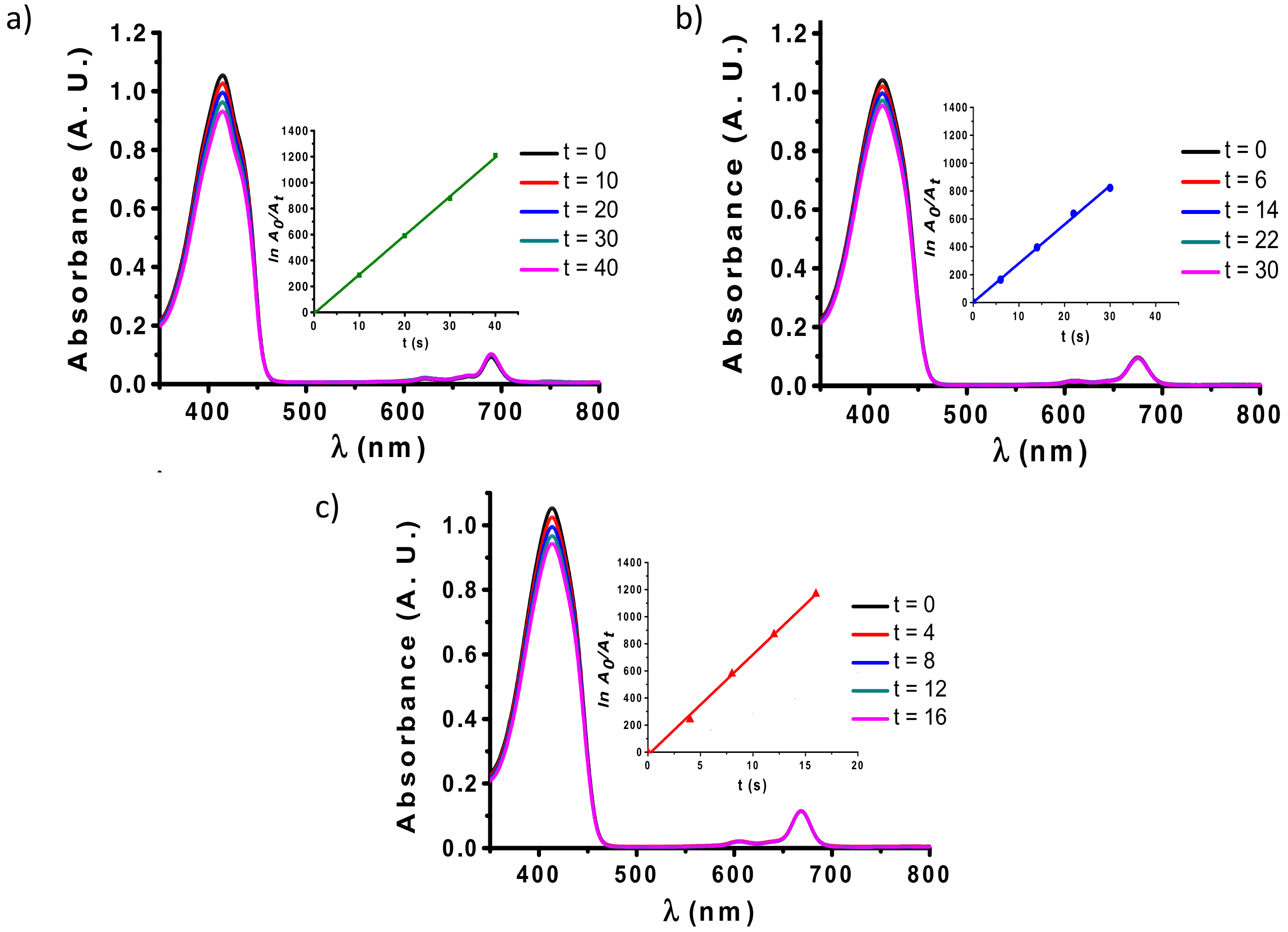

Figure S6. Time-dependent photobleaching of DBPF absorption in the presence of compound a) **1**, b) **2** and c) non-substituted ZnPc in DMF, which is directly related to the generation of $^1O_2$ induced by the PS irradiation.

2.4. Subcellular localization and phototoxicity experiments

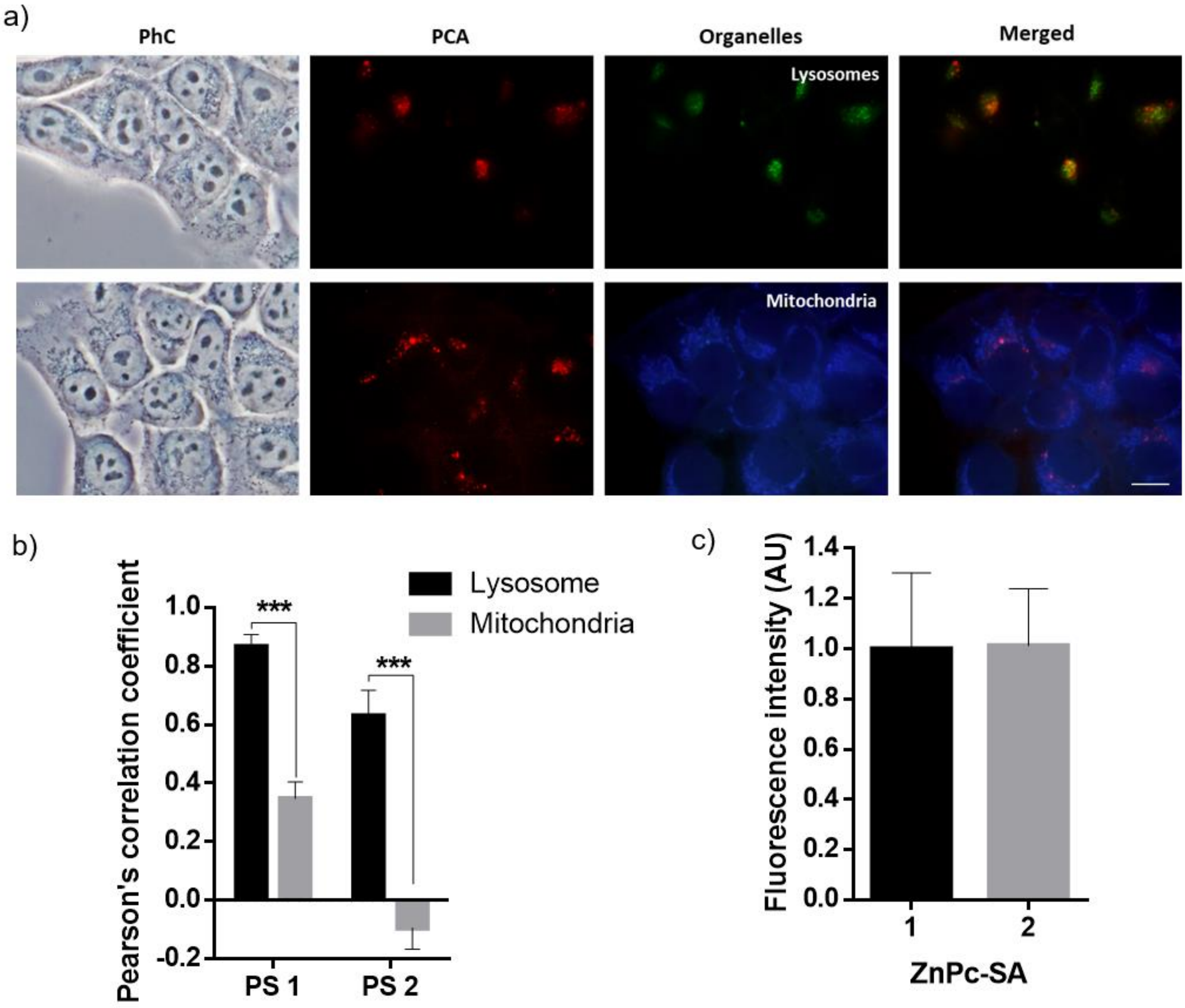

Figure S7. (a) Subcellular localization of ZnPc-SA **2** in SCC-13 cells after 18 h of incubation at 10 µM of PS. Phase contrast (PhC). Red fluorescence is from the PS **2**, green fluorescence is from lysosomes, and blue fluorescence is from mitochondria. The merged image shows **2** and organelles together. A blue (450–490 nm) exciting lamp was used for LysoTracker (top row of images) and UVA (360-370 nm) exciting lamp was used for MitoTracker (bottom row of images), while green (545 nm) exciting light was utilized for ZnPc-SA **2**. Scale bar 10 µm. (b) Pearson's correlation coefficient referred to the colocalization of PS and organelles. Each point corresponds to the mean value ± SD obtained from three independent experiments. ***P < 0.001. (c) Fluorescence intensity after incubation of the cells with PS **1** and PS **2** for 18 h at the same concentration, monitored by using the imageJ program. At least 100 SCC-13 cells were measured.

Table S1. Toxicity induced in different cell lines by incubation with **1** and **2** in the dark. Cell toxicity was evaluated by the MTT assay 24 h after treatment. Data are expressed as mean values obtained from three independent experiments ± standard deviation (SD).

Compound	Cells	Concentration [M]	Surviving fraction (%) ± SD
**1**	SCC-13	0.5×10^{-6}	101.6 ± 2.8
		10×10^{-6}	98.9 ± 4.0
**2**	A431	0.5×10^{-6}	103.5 ± 1.5
		10×10^{-6}	102.5 ± 2.9
	HeLa	0.5×10^{-6}	100.0 ± 1.6
		10×10^{-6}	98.4 ± 2.3
	SCC-13	0.5×10^{-6}	99.1 ± 0.7
		10×10^{-6}	95.0 ± 4.0

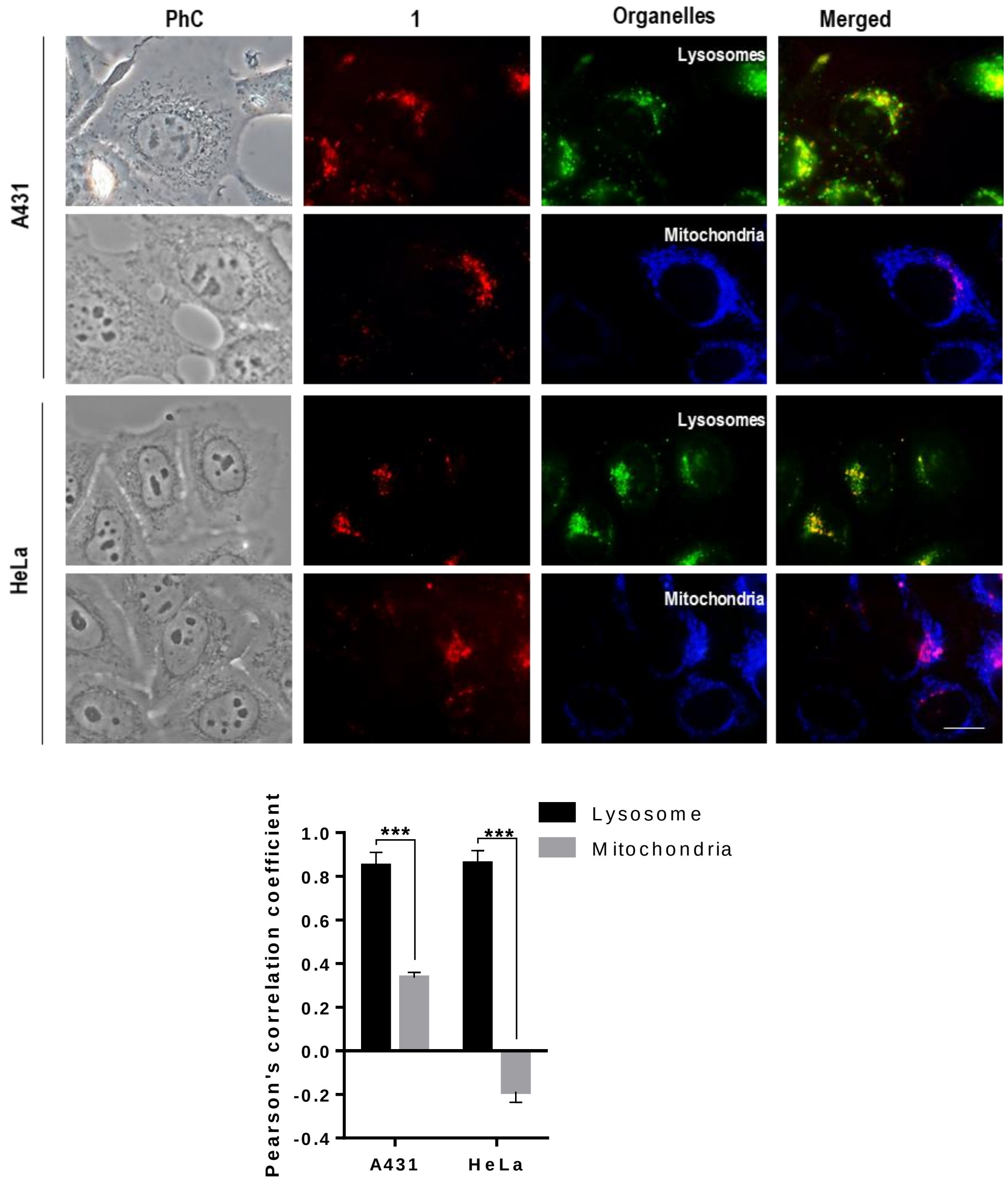

Figure S8. Subcellular localization of the ZnPc-SA **1** in A431 and HeLa cells after 18 h of incubation at 10 µM of PS. Phase contrast (PhC). Red fluorescence is from the ZnPc, green fluorescence is from lysosomes, and blue fluorescence is from mitochondria. The merged image shows the ZnPc-SA **1** and organelles together. A blue (450–490 nm) exciting lamp was used for LysoTracker (top row of images) and UVA (360-370 nm) exciting lamp was used for MitoTracker (bottom row of images), while green (545 nm) exciting light was utilized for the ZnPc-SA derivative. Scale bar 10 µm. Bottom: Pearson's correlation coefficient referred to the colocalization of PS and organelles. Each point corresponds to the mean value ± SD obtained from three independent experiments. ***P < 0.001.

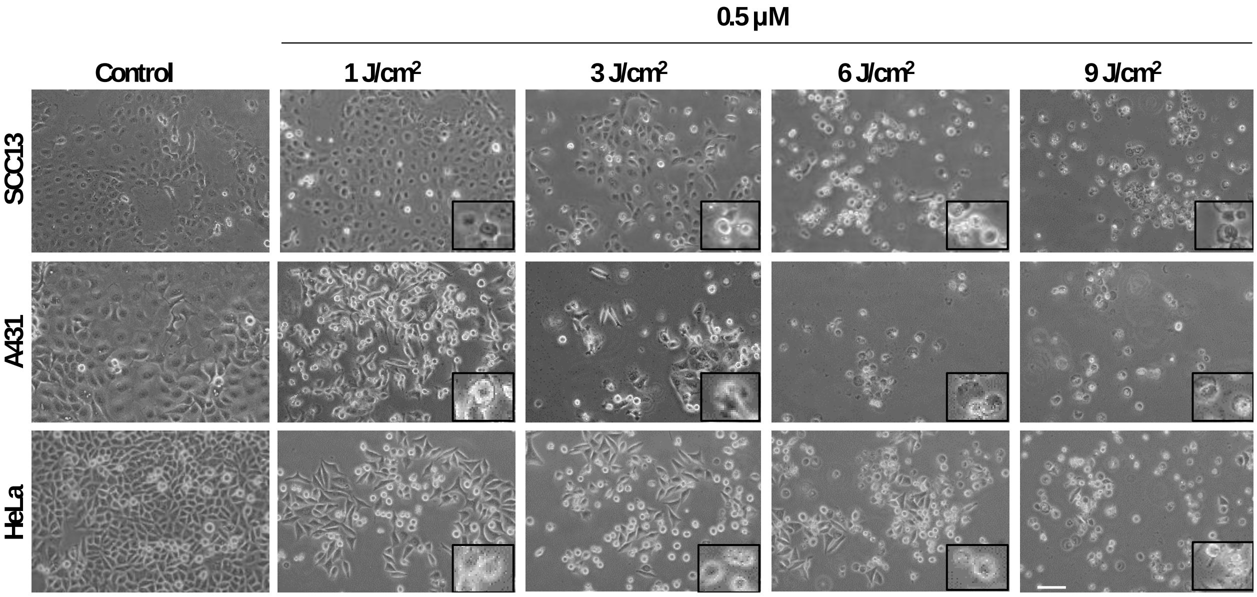

Figure S9. Morphological changes observed in SCC-13, HeLa, and A431cells 24 h after PDT with PS **1** (0.5 µM) upon irradiation with different red light doses (1–9 J/cm^2). Scale bar: 20 µm, details 5 µm.

2.5. Aggregation studies

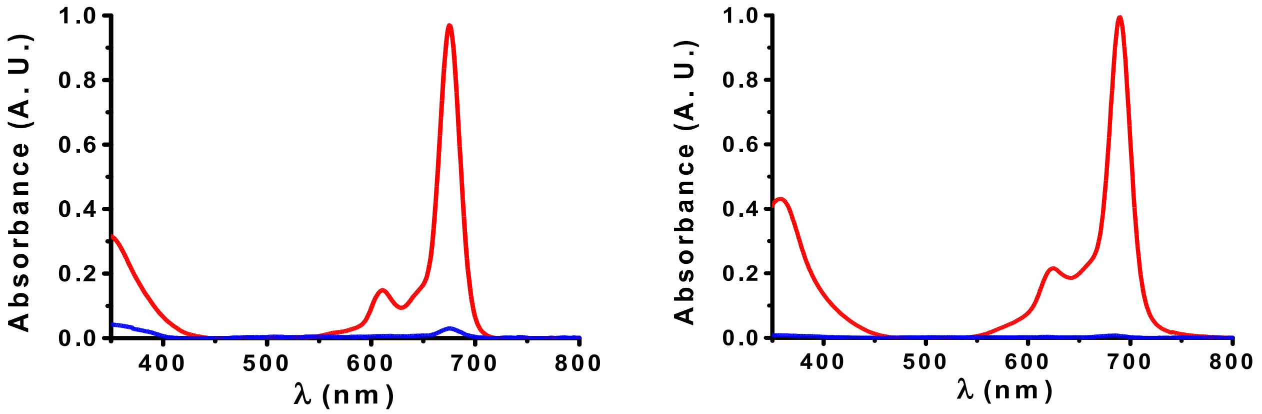

Figure S10. UV-Vis spectra recorded to calculate the partition coefficient in octanol (blue) and water (red) for compounds **1** (left) and **2** (right). The procedure for such calculations is indicated in section 1.4 of this SI.

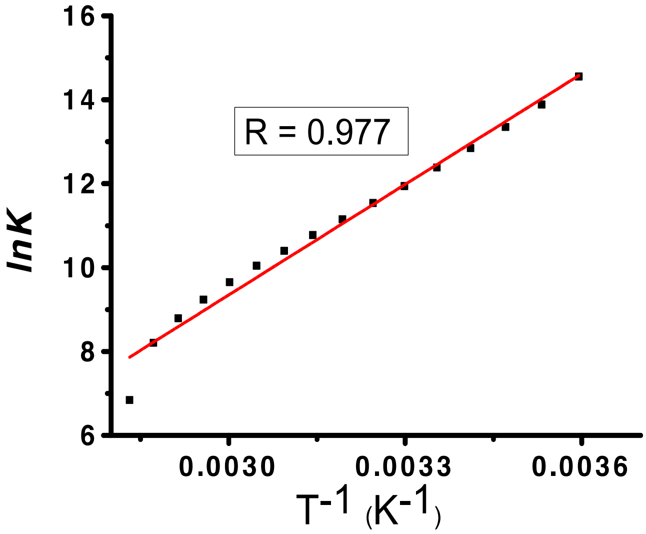

Figure S11. Van't Hoff analysis of the isodesmic supramolecular polymerization of compound **2** at 15 μM.

Table S2. Thermodynamic parameters of the isodesmic self-assembly process of **2** (DMF/PBS buffer 65:35) at 298 K.

Compound	[M]	λ (nm)	K_a M^{-1}	R^2	T_M K	DP_N	ΔH° [a] $kJmol^{-1}$	ΔS° [a] $Jmol^{-1}K^{-1}$	ΔG° [b] $KJmol^{-1}$	R
**2**	1.5×10^{-5}	645/691	238685.7435	0.998	303.72	1.57	-73.09±2.87	-141.56±9.18	-30.90±5.61	0.977

[a] Van't Hoff equation: $\ln K = -\Delta H^0 RT + \Delta S^0 R$; R = 8.3144621 J K^{-1} mol^{-1}. [b] Gibbs equation: $\Delta G^0 = \Delta H^0 - T\Delta S^0$.

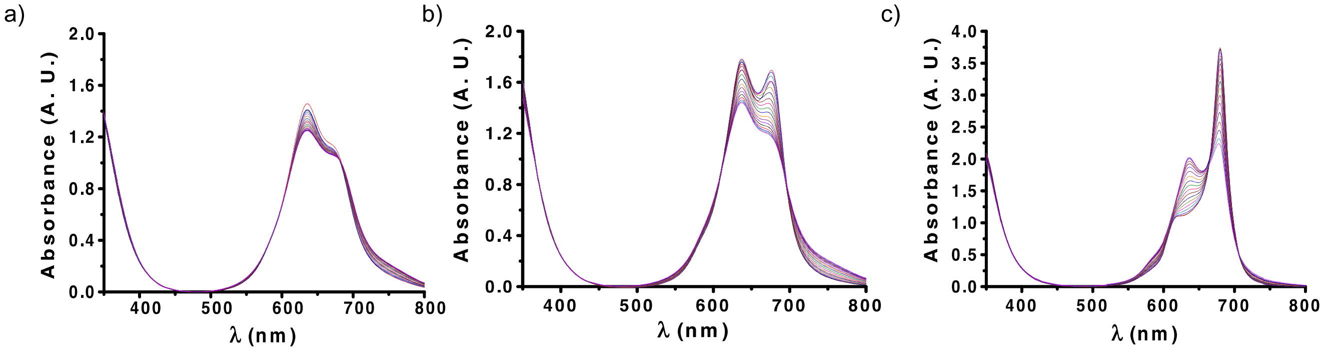

Figure S12. Cooling curves for the study of the supramolecular polymerization of compound **1** (50 µM), recorded at different proportions of DMF/PBS: (a) 10%, (b) 20% and (c) 40% DMF.

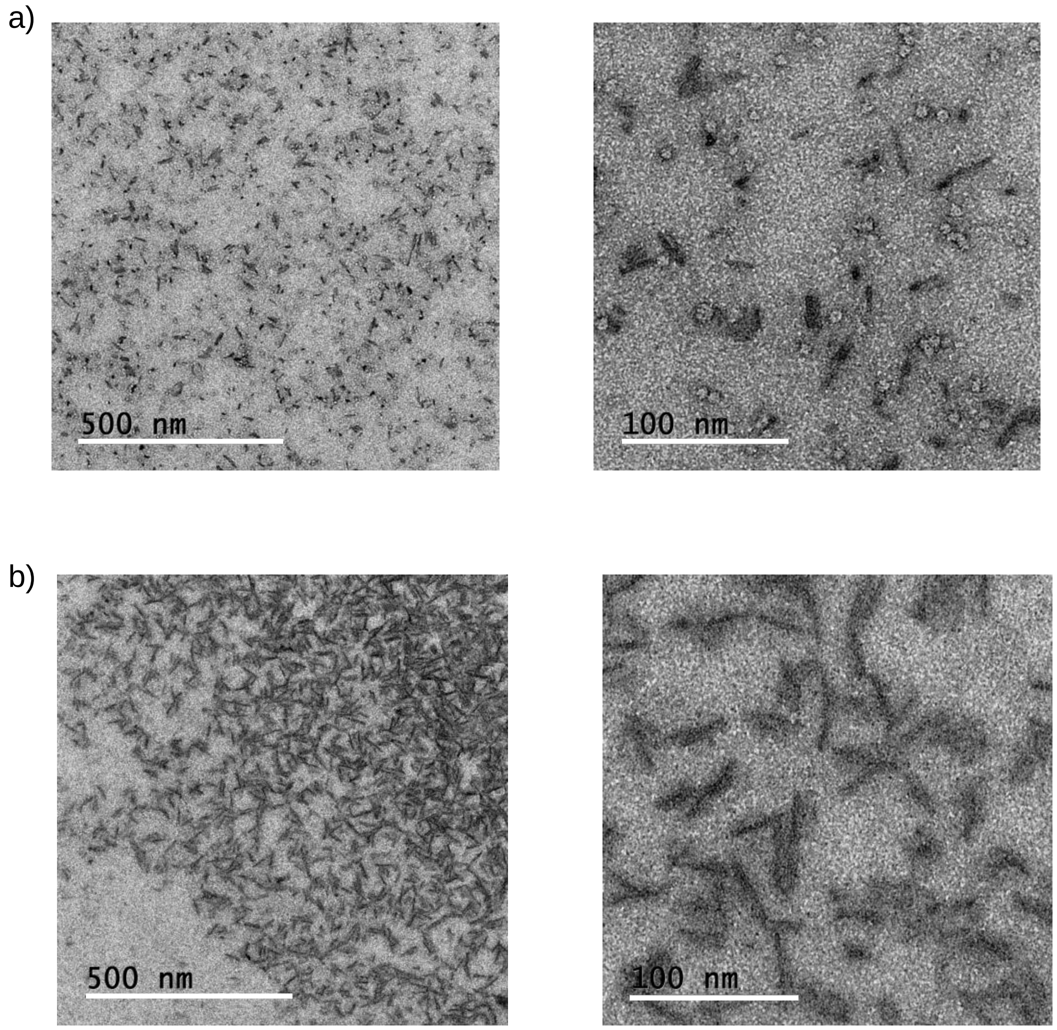

Figure S13. TEM micrographs of 50 µM (a) **1** and (b) **2**.

References

[1] A. Gheorghe, T. Chinnusamy, E. Cuevas-Yañez, P. Hilgers, O. Reiser, *Org. Lett.* **2008**, *10*, 4171-4174.
[2] S. Wolf, S. Warnecke, J. Ehrit, F. Freiberger, R. Gerardy-Schahn, C. Meier, *ChemBioChem* **2012**, *13*, 2605-2615.
[3] M. Weïwer, C.-C. Chen, M. M. Kemp, R. J. Linhardt, *Eur. J. Org. Chem.* **2009**, *2009*, 2611-2620.
[4] R. F. Enes, J.-J. Cid, A. Hausmann, O. Trukhina, A. Gouloumis, P. Vázquez, J. A. S. Cavaleiro, A. C. Tomé, D. M. Guldi, T. Torres, *Chem. Eur. J.* **2012**, *18*, 1727-1736.
[5] a) C. C. Leznoff, H. Lam, S. M. Marcuccio, W. A. Nevin, P. Janda, N. Kobayashi, A. B. P. Lever, *J. Chem. Soc. Chem. Comm.* **1987**, *9,* 1087; b) J. G. Young, W. Onyebuagu, *J. Org. Chem.,* **1990**, *55,* 2155.
[6] A. T. R. Williams, S. A. Winfield, J. N. Miller, *Analyst* **1983**, *108,* 1067.
[7] I. Scalise, E. N. Durantini, *Bioorg. Med. Chem.* **2005**, *13*, 3037-3045.
[8] S. Makhseed, A. Tuhl, J. Samuel, P. Zimcik, N. Al-Awadi, V. Novakova, *Dyes Pigm.* **2012**, *95*, 351-357.
[9] W. Spiller, H. Kliesch, D. Wöhrle, S. Hackbarth, B. Röder, G. Schnurpfeil, *J. Porphyr. Phthalocyanines* **1998**, *2*, 145-158.
[10] F. M. Engelmann, S. V. O. Rocha, H. E. Toma, K. Araki and M. S. Baptista, *Int. J. Pharm.*, **2007**, *329*, 12-18.
[11] a) M. M. J. Smulders, M. M. L. Nieuwenhuizen, T. F. A. de Greef, P. van der Schoot, A. P. H. J. Schenning, E. W. Meijer, *Chem. - Eur. J.* **2010**, *16*, 362-367; b) Z. Chen, A. Lohr, C. R. Saha-Möller, F. Würthner, *Chem. Soc. Rev.* **2009**, *38*, 564-584. (c) M. J. Mayoral, C. Rest, J. Schellheimer, V. Stepanenko, G. Fernández, *Chem. - Eur. J.* **2012**, *18*, 15607-15611.
[12] S. Ylä-Herttuala, J. B. Christensen, S. M. Moghimi, T. Torres-Cebada, P. N. Trohopoulos, P. Makinen, M. Ficker, L. Wu, M. Medel Gonzalez, “Nano-systems for therapy and/or diagnosis and/or therapy Monitoring and/or theranostics of disease” 2016, PCT/EP 16168476.6.
[13] M. Sommerauer, C. Rager and M. Hanack, *J. Am. Chem. Soc.* **1996**, *118*, 10085-10093.
